# Integrated single-cell chromatin and transcriptomic analyses of peripheral immune cells in patients with alopecia areata

**DOI:** 10.3389/fimmu.2025.1565241

**Published:** 2025-07-02

**Authors:** Jesus Gay-Mimbrera, Pedro Jesús Gómez-Arias, Pablo Álvarez-Heredia, Alexander Batista-Duharte, Irene Rivera-Ruiz, Macarena Aguilar-Luque, Miguel Juan-Cencerrado, Carmen Mochón-Jiménez, Álvaro Cebrián-García, Eloísa Andújar-Pulido, Mónica Pérez-Alegre, Alejandra Pera, Juan Ruano

**Affiliations:** ^1^ Inflammatory Immune-Mediated Chronic Skin Diseases Laboratory (GC26), Maimonides Biomedical Research Institute of Cordoba (IMIBIC)/University of Cordoba/Reina Sofia University Hospital, Córdoba, Spain; ^2^ Department of Dermatology, Reina Sofía University Hospital, Córdoba, Spain; ^3^ Immunology and Allergy Group (GC01), Maimonides Biomedical Research Institute of Cordoba (IMIBIC)/University of Cordoba/Reina Sofia University Hospital, Cordoba, Spain; ^4^ Genomic Unit, Andalusian Molecular Biology and Regenerative Medicine Center (CABIMER), CSIC-University of Seville-University Pablo de Olavide, Seville, Spain; ^5^ Department of Cell Biology, Physiology and Immunology, University of Cordoba, Cordoba, Spain; ^6^ Department of Medicine, University of Córdoba, Córdoba, Spain

**Keywords:** Alopecia areata, severity, peripheral blood, immune cells, single-cell RNA sequencing, chromatin accessibility, immune regulation, pseudotime

## Abstract

**Introduction:**

Alopecia areata (AA) is an autoimmune disorder characterized by non-scarring hair loss ranging from mild, self-limiting episodes to severe and chronic forms. While prior research has primarily focused on lesional skin, the contribution of systemic immune cells remains underexplored.

**Methods:**

We performed integrated single-cell RNA sequencing (scRNA-seq) and single-cell assay for transposase-accessible chromatin sequencing (scATAC-seq) on peripheral blood mononuclear cells (PBMCs) from patients with mild and severe AA, as well as healthy controls. A total of 32,453 high-quality cells were analyzed across 36 immune cell subtypes.

**Results:**

In AA patients, especially those with severe disease, we observed increased transcriptional heterogeneity, cytokine and chemokine pathway activation, and upregulation of antigen-presentation machinery enriched in TH1, TH2, and TH17 signatures. Chromatin accessibility profiling revealed 42,248 significant peaks with pronounced epigenetic remodeling in *CD14* ^+^ monocytes, NK cells, and *CD8* ^+^ T cells. Mild AA showed early immune regulatory failure, with elevated exhaustion markers in double-negative T cells and increased apoptosis in myeloid populations. Pseudotime and transcription factor analyses indicated altered differentiation trajectories, and inferred cell-cell communication networks highlighted monocytes, NK cells, and memory T cells as key signaling hubs.

**Discussion:**

Our results provide the first integrated single-cell chromatin and transcriptomic map of peripheral immune dysregulation in AA. These findings uncover systemic alterations associated with disease severity and identify candidate pathways for immune modulation and therapeutic targeting.

## Introduction

Alopecia areata (AA) is a common, unpredictable, immune-mediated hair loss disorder with varied clinical presentations (1). Managing AA poses significant challenges in achieving long-term control, efficacy, and safety (2). Severe forms, such as alopecia totalis (AT) and alopecia universalis (AU), frequently relapse, and their underlying mechanisms remain poorly understood (3, 4). Predicting the clinical course of AA is difficult due to limited understanding of the driving immunological mechanisms (5–12). Emerging evidence suggests the involvement of systemic factors beyond local follicular inflammation (13, 14), including associations with other immune-mediated diseases (15, 16) and environmental influences (17, 18). Severe AA has also been linked to heightened systemic inflammation and increased cardiovascular risk (19, 20).

In healthy skin, anagen-phase hair follicles (HFs) are protected from immune-mediated damage by a specialized form of immune privilege (IP), which extends from the follicular bulge—where keratinocyte stem cells reside—down to the bulb (21). During anagen, the HF undergoes intense growth, characterized by high mitotic activity of keratinocytes and melanocytes in the bulb. This creates an immunologically active environment, increasing the likelihood of presenting neoantigens or self-antigens, particularly those derived from melanocytes. The increased perifollicular vascularization during this phase also facilitates the entry of circulating immune cells—including potentially autoreactive lymphocytes—into the follicular microenvironment.

This immune protection is maintained by both passive and active mechanisms. These include the downregulation of MHC class I and II molecules, the secretion of immunosuppressive factors such as α-melanocyte-stimulating hormone (*α-MSH*), transforming growth factor-beta 1 and 2 (*TGF-β1*, *TGF-β2*), interleukin-10 (*IL-10)*, and cortisol, as well as the expression of non-classical MHC molecules like *HLA-E* and *HLA-G* (22). Additionally, antigen-presenting cells (APCs), such as Langerhans cells, are excluded from the follicular epithelium. The absence of lymphatic vessels in the lower follicle region further limits antigen drainage and immune cell trafficking. Together, these mechanisms reinforce the immune-privileged status of the HF.

In AA, this equilibrium is disrupted—either by environmental insults or intrinsic immune regulatory defects—leading to the collapse of HF IP, which represents a key initiating event in disease pathogenesis (21, 23). This breakdown results in the upregulation of MHC class I and II molecules by follicular epithelial cells, facilitating the presentation of melanocyte-associated autoantigens to *CD8* ^+^ and *CD4* ^+^ T cells (24). Interferon-gamma (*IFN-γ*) acts as a central mediator by activating the *JAK*/*STAT* signaling pathway, thereby amplifying antigen presentation and inducing the expression of IFN-inducible chemokines (*CXCL9*, *CXCL10*, *CXCL11*) and interleukin-15 (*IL-15*) (25, 26). These mediators promote the recruitment of autoreactive *CD8* ^+^ T cells and *NKG2D* ^+^ natural killer (NK) cells to the HF bulb, where they exert cytotoxic effects against follicular structures (27). This immune infiltration establishes a self-perpetuating inflammatory loop that sustains immune activation and follicular damage, ultimately driving the premature transition of anagen HFs to the telogen phase and resulting in hair shedding.

Increasing evidence suggests that the immunopathology of AA, initially localized to the HF, is also mirrored in the systemic immune compartment. Peripheral blood analyses in AA patients have demonstrated altered frequencies of immune cell subsets, including increased proportions of cytotoxic *CD8* ^+^ T cells and NK cells, along with reduced numbers or impaired function of regulatory T cells (Tregs) (28, 29). These systemic alterations parallel the immune infiltrates observed in lesional skin, supporting the hypothesis that follicular immune dysregulation leaves a measurable systemic footprint (25, 29). Cytokine profiling in blood has consistently shown elevated levels of *IFN-γ*, *IL-15*, *IL-2*, and *CXCL10*—key mediators of both *JAK*/*STAT* signaling and IP collapse in the follicular epithelium (30–32). Notably, these changes correlate with disease severity, as patients with extensive or chronic forms of AA display stronger Th1/*IFN*-driven signatures and broader immune activation across both innate and adaptive compartments (25, 33).

The transition from patchy to extensive disease may involve the loss of regulatory circuits and progressive amplification of proinflammatory loops, supported by evidence of Treg dysfunction in both tissue and blood (28, 34). Chronicity and treatment resistance have also been associated with increased expression of exhaustion markers (e.g., *PD-1*, *CTLA-4*) on circulating T cells, persistent type I/II *IFN* signatures, and sustained cytotoxic activity by memory *CD8* ^+^ T cells (25, 32). Recent transcriptomic and epigenomic studies confirm that many of the pathways activated in lesional skin—such as antigen presentation, cytotoxicity, and *IFN* signaling—are also reflected in blood immune cells, particularly in patients with severe or refractory disease (25, 28, 32).

We hypothesize that severe, chronic AA involves epigenetic reprogramming of circulating immune cells, contributing to systemic dysregulation (35–39). Identifying disease-driving cell populations and transcription factor (TF)-controlled gene programs may help explain phenotypic heterogeneity and support the development of prognostic tools and personalized therapies (40).

Bulk transcriptomic approaches, such as microarrays or bulk RNA sequencing, are limited by their inability to resolve transcriptional heterogeneity across distinct immune cell types (9, 39–44). By averaging gene expression across diverse cell populations, these methods obscure cell-specific disease signatures that may be critical to understanding autoimmune pathogenesis. Single-cell technologies—such as single-cell RNA sequencing (scRNA-seq) and single-cell assay for transposase-accessible chromatin using sequencing (scATAC-seq)—overcome these limitations and have enabled deeper investigation of immune-mediated skin diseases, including atopic dermatitis (45–49), psoriasis (50–52), prurigo nodularis (53, 54), and mycosis fungoides (55–58). While scRNA-seq provides high-resolution insights into cellular composition and gene expression, scATAC-seq allows for the study of chromatin accessibility and transcriptional regulation (59, 60).

Therefore, in this study we performed integrated scRNA-seq and scATAC-seq on PBMCs of AA patients (mild and severe) and healthy controls, aiming to characterize systemic immune dysregulation associated with disease severity.

## Materials and methods

### Patient selection and sample collection

**Figure 1:**
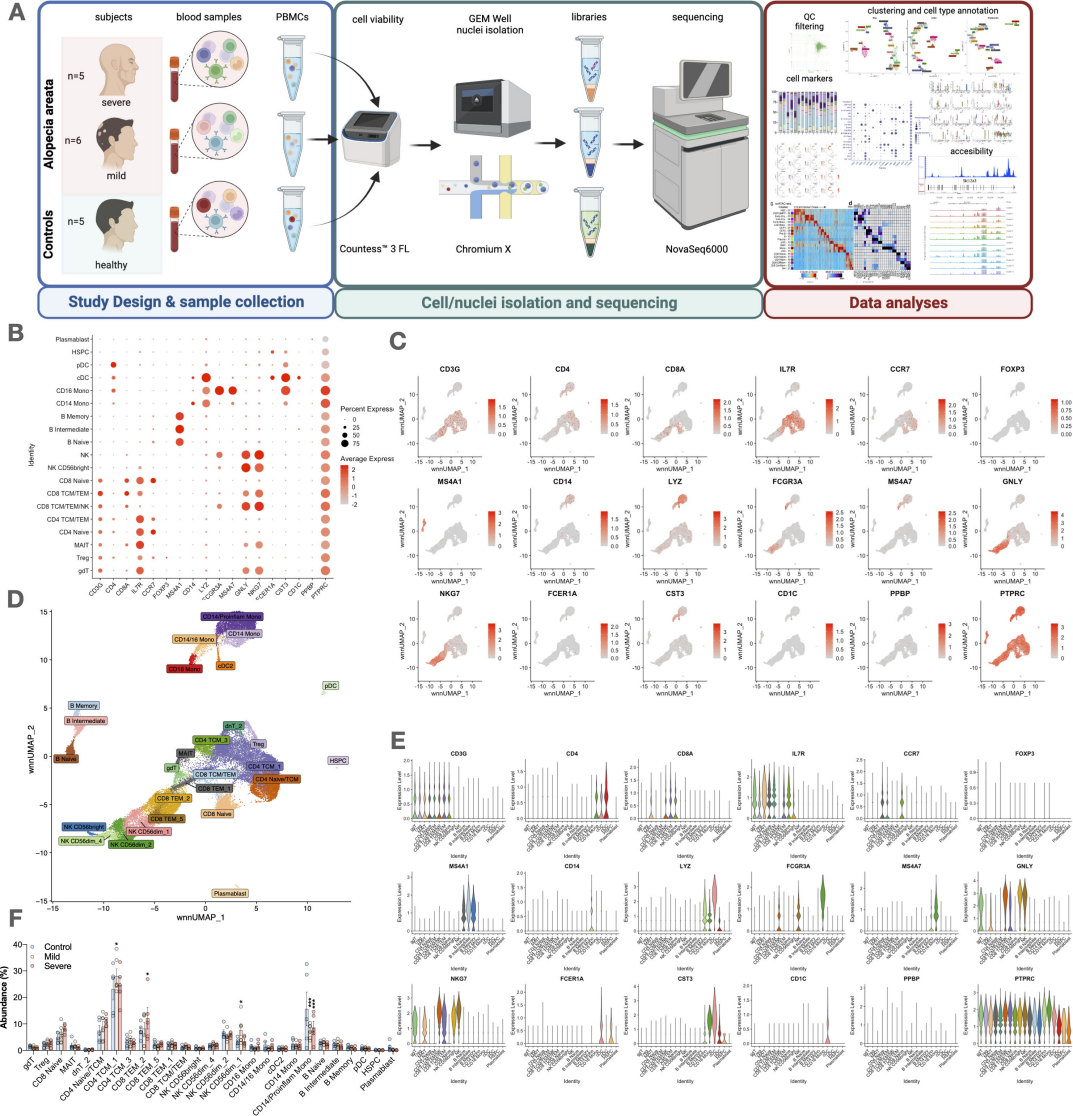
Study design, cell type abundance, and marker expression in alopecia areata patients and controls. **(A)** Overview of the study design, from blood collection to cell/nuclei isolation, sequencing (scRNA-seq/scATAC-seq), and downstream analysis. **(B)** Dot plot showing expression of key markers across immune cell types. **(C)** UMAP feature plots illustrating distribution of representative genes (e.g., CD3G, CD4, FOXP3). **(D)** UMAP clustering of major immune subsets: T cells (CD3+), CD4+ (CCR7, IL7R), CD8+ (CD8A), Tregs (FOXP3), monocytes (CD14, FCGR3A), NK cells (GNLY, NKG7), B cells (CD19, MS4A1), plasmablasts (CD38), dendritic cells (FCER1A, CST3), HSPCs (PTPRC). **(E)** Violin plots of selected marker genes across cell types. **(F)** Dot plot comparing expression levels and cell fractions by disease group (control, mild AA, severe AA). Statistical analysis is denoted by asterisks: ***p < 0.001, *p < 0.05, and “ns” non-significant difference.

We collected fresh peripheral blood samples from patients with alopecia areata (AA), excluding those with ophiasis or sisaipho patterns, and enrolled age-, sex-, and ethnicity-matched healthy controls through the outpatient clinic of the Department of Dermatology at Reina Sofía University Hospital, Córdoba, Spain (**Figure 1A**).

A total of 35 individuals were enrolled: 12 patients with severe AA, 11 with mild AA, and 12 healthy controls. All were included in the flow cytometry analyses. From this group, a subset of 16 samples—comprising 5 severe AA, 6 mild AA, and 5 controls—was selected for single-cell RNA sequencing (scRNA-seq) and single-cell chromatin accessibility profiling (scATAC-seq) based on stringent quality control criteria and sequencing performance.

Eligible participants were aged ≥18 years and had a confirmed clinical diagnosis of AA. Patients with other immune-mediated diseases were excluded, except for those with clinically stable Hashimoto’s thyroiditis. These individuals were included only if they had not received systemic immunomodulatory or corticosteroid therapy in the 8 weeks prior to sampling. All patients were free from systemic immunosuppressive treatments—including corticosteroids, methotrexate, cyclosporine, or JAK inhibitors—for at least 8 weeks. In addition, topical therapies (e.g., corticosteroids, minoxidil) were discontinued at least 4 weeks before sample collection to minimize pharmacological effects on circulating immune cells. None of the participants were receiving systemic immunosuppressants at the time of sampling (**Table 1**).

**Table 1:** Patient baseline characteristics at the time of sampling.

Subject ID	Type	Comorbidities	Age (y)	Sex	Race	Disease duration	SALT	Ongoing treatment	Previous treatments	Evolution 1 year
AA032	AT	Hypothyroidism, hypercholesterolemia	49	F	White	40 y	100%	None	PRP DPCP DXM Mini-pulses	No changes
AA037	AT	Vulgar Warts, Psoriasiform lesions, IBD	40	F	White	13 y	100%	None	Topical corticosteroids DXM Mini-pulses Infliximab Ustekinumab Adalimumab	No changes
AA040	AT	Depressive disorder	46	F	White	33 y	100%	None	Topical corticosteroids Latanoprost and bimatoprost Cyclosporine Hydroxychloroquine	No changes
AA042	AT	None	23	F	White	18 y	100%	None	DPCP DXM Mini-pulses Topical corticosteroids Methotrexate, Ritlecitinib Cyclosporine	No changes
AA049	AU	Hypothyroidism, Hypertension	52	F	White	16 y	100%	None	DXM Mini-pulses Cyclosporine DPCP	No changes
AA033	AA MP	Renal lithiasis, Dyslipidemia	47	M	White	6 y	4%	None	Topical and systemic corticosteroids	Total recovery
AA034	AA MP	None	41	M	White	6 y	31%	None	Biotine	Partial improvement
AA035	AA MP	None	50	F	White	3 m	19%	None	None	Total recovery
AA038	AA SP	Hypothyroidism, hyperglycemia	27	M	White	4 m	3%	None	None	Single patch relapsing
AA041	AA MP	Hypothyroidism	41	F	White	2 m	5%	None	None	Multiple patch relapsing
AA043	AA SP	None	45	M	White	8 m	1%	None	Topical corticosteroids	Single patch relapsing
CN205	HC	None	40	M	White	NA	NA	NA	NA	NA
CN206	HC	None	36	F	White	NA	NA	NA	NA	NA
CN209	HC	None	21	M	White	NA	NA	NA	NA	NA
CN210	HC	Nome	41	F	White	NA	NA	NA	NA	NA
CN212	HC	None	43	F	White	NA	NA	NA	NA	NA

Patients with clinically stable Hashimoto’s thyroiditis were included if not receiving systemic immunomodulatory treatment. All participants were free from systemic immunosuppressive therapy for ≥8 weeks and had discontinued topical treatments (e.g., corticosteroids, minoxidil) at least 4 weeks before blood sampling. AA, Alopecia Areata; DPCP, Diphenylcyclopropenone (also known as diphencyprone); DXM, Dexamethasone; HC, Healthy Control; IBD, Inflammatory Bowel Disease; MP, Multiple Patches; PRP, Platelet-Rich Plasma; SP, Single Patch; NA, Not Applicable..

### Severity criteria for AA

Severity was assessed using the Severity of Alopecia Tool (SALT) and characteristics of the most recent flare (61). Patients were classified into two groups: mild–moderate AA, defined as <50% scalp involvement and disease duration under one year; and severe AA, defined as SALT ≥50%, duration of one year or longer, or the presence of AT or AU.

*Single-cell isolation, nuclei suspension preparation, and Single-Cell workflow*

Human peripheral blood mononuclear cells (PBMCs) were isolated by Ficoll-Paque density gradient centrifugation and cryopreserved 2x106 cells per sample. Cell viability was 95% or greater for all samples. PBMCs were quickly thawed at 37°C in a water bath and washed with culture medium RPMI 1640 supplemented with 10% FBS. DNase treatment was done before the nuclei isolation. The nuclei isolation was conducted with an RNase inhibitor. Nuclei suspension was filtered with a 40µm cell strainer and counted in a Countess 3 FL Automated Cell Counter (Invitrogen, USA); all samples had <5% live cells. High-quality nuclei were checked by 60x brightfield microscopy. The targeted number of nuclei was 5000 nuclei per sample. Finally, single-cell chromatin accessibility and gene expression profiling was performed using the Chromium Next GEM Single Cell Multiome ATAC + Gene Expression Kit (10x Genomics, USA), in accordance with the manufacturer’s protocol.

### scRNA-seq and scATAC-seq library preparation and sequencing

scRNA-seq libraries were prepared according to the manufacturer’s instructions using the Chromium Single Cell 3′ Reagent Kits v2 Chemistry (10x Genomics, USA), and sequenced in multiplex on the NovaSeq 6000 platform (Illumina, USA) at Cabimer’s Genomics Core Facility. Raw sequencing data were processed with the Cell Ranger ARC pipeline (v2.0.0; 10x Genomics, USA) for FASTQ generation, demultiplexing, alignment to the GRCh38 human reference genome, and generation of gene-barcode matrices in Linux, following the manufacturer’s guidelines. This included: (1) an ATAC matrix computation step involving barcode processing, read trimming, read alignment, duplicate marking, peak calling, and peak-barcode matrix generation using either the mm10 mouse or GRCh38 human reference genome; and (2) a gene expression (GEX) matrix computation step comprising read trimming, genome and transcriptome alignment, UMI correction, and UMI counting; followed by joint cell calling. Downstream secondary analyses for ATAC and GEX data included dimensionality reduction, clustering, peak annotation, transcription factor analysis, differential expression analysis, differential accessibility analysis, and feature linkage, as described above, using the Seurat (Satija Lab, USA), Signac (Stuart Lab, USA), and ArchR (Greenleaf Lab, USA) toolkits.

### Quality report of scRNAseq analysis for circulating immune cells in AA

Read quality control was performed using FastQC (Babraham Bioinformatics, UK) for each FASTQ file, and results were aggregated using MultiQC (Ewels et al., Sweden). Reads were aligned to the human reference genome (GRCh38) using Cell Ranger ARC v2.0.2 (10x Genomics, USA). Raw count matrices were imported into R v4.1.2 (R Core Team, Austria) and analyzed using Seurat v4.3.0.1 (Satija Lab, USA). Dead cells were excluded based on quality control thresholds: fewer than 800 RNA features, fewer than 3,000 ATAC features, or more than 20% mitochondrial gene content.

### Dimension reduction and cell clustering

RNA datasets were normalized and variance-stabilized using SCTransform, as implemented in Seurat v4.3.0.1 (Satija Lab, USA). Integration was performed by selecting 3,000 highly variable features and identifying integration anchors based on SCTransform normalization. ATAC datasets were processed using the standard pipeline provided by Signac v1.10.0 (Stuart Lab, USA). Integration of ATAC data involved quantifying multiome peaks to identify common features, merging datasets, finding integration anchors, and integrating the Latent Semantic Indexing (LSI) embeddings. A weighted combination of RNA and ATAC-seq modalities was achieved using the WNN approach. Dimensionality reduction was carried out using Uniform Manifold Approximation and Projection (UMAP) on the first 30 dimensions. Clustering was performed using the Smart Local Moving (SLM) algorithm on the WNN graph, yielding 36 annotated clusters based on canonical cell type marker scores defined via Azimuth v0.4.6 (Satija Lab, USA). Data visualization was performed using internal plotting functions in Seurat.

### Differential gene expression and functional enrichment analyses

Differentially expressed genes (DEGs) were identified using the Wilcoxon rank-sum test and logistic regression models, as implemented in Seurat (Satija Lab, USA). Functional annotation was performed using DAVID Bioinformatics Resources 2021 (Laboratory of Human Retrovirology and Immunoinformatics, NIH, USA). Pathway enrichment analysis was conducted with GeneCodis 4 (University of Granada and CIPF, Spain), using curated pathway databases including Gene Ontology (GO), Kyoto Encyclopedia of Genes and Genomes (KEGG), PANTHER, Reactome, and WikiPathways (62).

### Pseudotime analyses

To reconstruct cellular differentiation dynamics, we applied established computational frameworks tailored for scRNA-seq data. Pseudotime trajectories were inferred using the standard pipeline of Slingshot v2.2.1, enabling robust modeling of lineage progression and temporal ordering of cells. This method was selected for its capacity to resolve both intra- and inter-lineage developmental paths, facilitating the identification of convergent and divergent differentiation events across cell types. Through this approach, we gained insight into the hierarchical structure of immune-mediated responses and the sequence of cellular transitions specific to AA.

DEG analysis was subsequently performed along the inferred trajectories, using appropriate statistical thresholds with FDR correction to ensure rigor. This yielded a curated list of genes characterized by dynamic expression changes along pseudotime, offering clues into their potential regulatory roles in lineage specification and differentiation.

To comprehensively interrogate these dynamics, we performed four distinct pseudotime-based comparisons, each capturing a unique facet of the differentiation process:

1. Pseudotime Association Across All Trajectories (1a): Genes whose expression levels varied along pseudotime, independent of the final lineage. These genes may be involved in general differentiation programs.
2. Start-to-End Trajectory Comparison (1b): Genes differentially expressed between the initial and terminal stages of each trajectory. This analysis highlights regulators of commitment and terminal maturation.
3. Lineage-Specific Terminal State Association (2a): Genes that distinguish the end points of the two major lineages, revealing molecular signatures that define mature cell fates.
4. Trajectory-Specific Expression Dynamics (2b): Genes showing differential expression patterns at any point along the two trajectories. These likely contribute to pathway divergence and cell fate determination.

### Cell communication networks

Intercellular communication is essential for maintaining tissue homeostasis and orchestrating physiological responses. To dissect the complex signaling interactions among cell populations, we employed CellChat v1.6.1 (Jin et al., USA), an advanced computational tool for inferring and analyzing cell-cell communication networks from single-cell transcriptomic data. We applied the 10% truncated mean method to calculate average gene expression within each cell group, a strategy that minimizes the influence of outliers and preserves the robustness of downstream analyses.

CellChat enabled the reconstruction of a comprehensive signaling landscape, revealing potential crosstalk and pathway activity between immune and non-immune cell populations. This analysis provided valuable insights into the regulatory mechanisms driving the cellular phenotypes observed in AA, highlighting key ligand-receptor interactions and signaling hubs that may represent novel therapeutic targets.

### Assay for transposase-accessible chromatin using sequencing and assignment of candidate transcription factors

ATAC-seq data were processed using the standard pipeline of Signac v1.10.0 (Stuart Lab, USA). Dataset integration was achieved by quantifying multiome peaks to identify shared chromatin accessibility features, followed by dataset merging, anchor identification, and integration of Latent Semantic Indexing (LSI) embeddings. A WNN approach was employed to combine chromatin accessibility (ATAC) and gene expression (RNA) modalities, providing a unified multimodal representation of the data.

Differential chromatin accessibility across experimental conditions was assessed using a logistic regression framework, and genomic annotation of ATAC peaks was performed by assigning each peak to its nearest gene using Signac functions. To identify potential regulatory mechanisms, we performed TF activity analysis using SCENIC (Single-Cell rEgulatory Network Inference and Clustering), enabling the inference of gene regulatory networks and the identification of key TFs driving cell-state transitions in PBMCs.

### Immunophenotyping by flow citometry

Cryopreserved PBMCs were thawed, washed with phosphate-buffered saline (PBS), and stained with fluorochrome-conjugated monoclonal antibodies targeting surface markers to identify innate (monocytes, natural killer [NK] cells) and adaptive (T and B cells) immune populations (see **Supplementary Table S2** for antibody panel). Sample acquisition was performed using a 20-parameter LSRFortessa SORP flow cytometer (BD Biosciences, USA), and data were analyzed with FlowJo v10.10 (BD, USA). Cell subset abundances across disease conditions were modeled using a Poisson Generalized Linear Model (GLM), adjusting for potential confounders including sex, age, and SALT score.

### Statistical analyses

All statistical analyses and data visualizations were performed using R (R Core Team, Austria) and Python programming environments, employing relevant packages and libraries. P-values obtained from all statistical models were corrected for multiple testing using the False Discovery Rate (FDR) method. Adjusted P-values < 0.05 were considered statistically significant.

### Data and code availability

The scRNA-seq data generated and analyzed during this study have been deposited in the Gene Expression Omnibus (GEO) under the accession number GSE277469. Additional data supporting the findings of this study, as well as custom code used for data processing and analysis, are available from the corresponding author upon reasonable request.

## Results

### Sequencing, mapping, and cell metrics confirm robustness of circulating PBMC dataset

The analysis included 32,453 quality-filtered cells, with each sample yielding an average of 3,269 cells (range: 1,426 to 4,748) and an average of 54,590 raw reads per cell (range: 17,055 to 122,594). These data correspond to a selected subset of 16 high-quality samples (5 severe AA, 6 mild AA, and 5 controls) from the broader study cohort, which included 12 severe AA, 11 mild AA, and 12 control participants. We obtained 8,004 cells from controls, 7,807 from mild AA patients, and 16,642 from severe AA patients (**Figure 1B**).

#### Sequencing quality and mapping metrics

Sequencing depth was adequate across all samples, with a mean of 187.8 million read pairs (range: 152.1–211.5M). The average Q30 base percentage was 93.8%, with Read 1 and Read 2 achieving 96.0% and 95.2%, respectively. Valid barcode and UMI rates were consistently high, averaging 98.1% and 99.9%, respectively, indicating reliable capture of single-cell information. Duplicate read rates ranged from 75.2% to 100.2%, reflecting some variability in library complexity. TSO rates varied from 2.5% to 9.0% across samples.

Genome mapping metrics were also consistent, with an average of 97.5% of reads mapped to the genome and 93% mapped confidently. On average, 54.4% of reads aligned to exonic regions, 32.2% to intronic, and 29.8% to intergenic regions. Transcriptome alignment rates averaged 70.5%, and antisense mapping was low (11.8%). Most samples showed a high proportion of confidently mapped read pairs (avg. 89.5%), although a few samples (e.g., CON210, 89.0%) were slightly lower. Unmapped read fractions were low overall, with a few exceptions (e.g., ARE049, 3.4%).

#### Cell and fragment quality metrics

The average estimated number of cells per sample was 3,269 (range: 1,426–4,748). Mean raw reads per cell were 54,590 (range: 17,055–122,594), with a consistent transcriptomic read fraction (avg. 68.2%). Median UMI counts and gene numbers per cell were 2,212 and 1,235, respectively. The average fraction of high-quality fragments was ~50%, with lower values in some samples (e.g., ARE038, 35.7%).

#### Mapping and alignment metrics

Reads mapped to the genome were consistently high across samples, with an average of 97.5%. Confident genome alignment averaged 93%, with 54.4% of reads mapping to exonic regions, 32.2% to intronic regions, and 29.8% to intergenic regions. Approximately 70.5% of reads aligned confidently to the transcriptome. Antisense mapping was low (mean: 11.8%), indicating correct strand orientation in the majority of reads.

The proportion of confidently mapped read pairs averaged 89.5%, with minor variability across samples (e.g., CON210: 89.0%). Most samples showed low fractions of unmapped reads; however, ARE049 had a slightly elevated rate (3.4%). Non-nuclear reads, considered technical noise, remained minimal across samples.

The number of captured cells varied between 1,426 (CON210) and 7,478 (CON209), with corresponding variability in sequencing depth per cell. Some samples (e.g., CON210) exhibited high read pairs per cell (119,894), potentially reflecting lower cell recovery or deeper sequencing. The average fraction of high-quality fragments was approximately 50%, with some samples, such as ARE038, showing lower values (35.7%).

#### ATAC-seq targeting quality metrics

The number of peaks per sample varied, with a maximum of 91,936 in CON209, possibly indicating sample-specific overamplification. The fraction of genome in peaks was relatively consistent across samples. Transcription Start Site (TSS) enrichment scores were generally acceptable, although some samples fell below the recommended threshold of 4. High-quality fragment fractions also showed variability, with some samples (e.g., CON211, 30.3%) showing reduced targeting efficiency.

### Comprehensive cell type annotation reveals altered peripheral monocyte and lymphocyte subsets in AA

Using 18 key markers and the most prominently expressed genes in each cluster, we identified 19 primary cell types through WNN graph-based clustering with a supervised learning method (**Figures 1B, C, E**). Each cluster was named according to canonical cell type markers assigned via Azimuth version 0.4.6.

**Figure 2:**
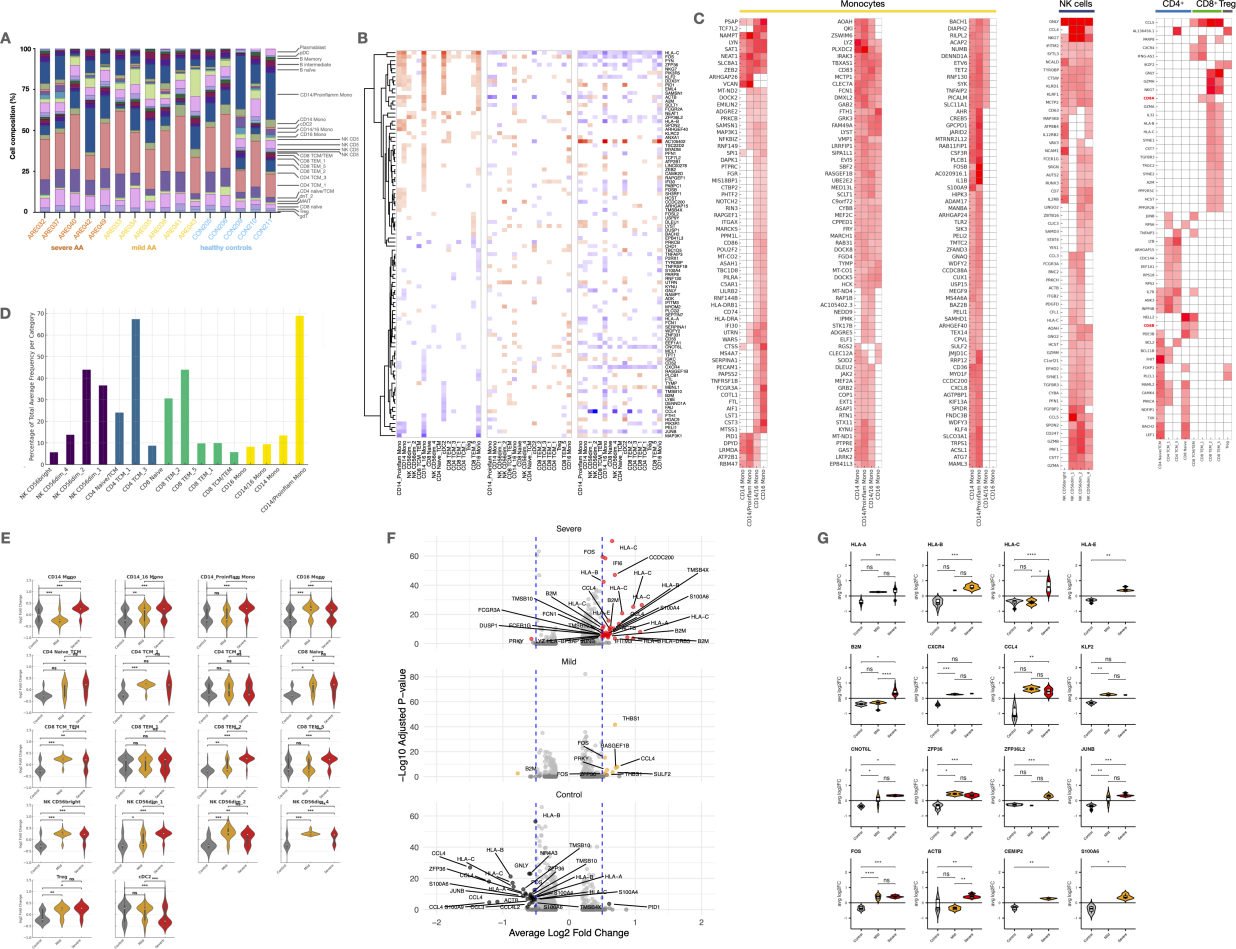
Cell type-specific expression patterns and differential gene expression in Alopecia. **(A)** Stacked bar plots showing immune cell proportions across groups. **(B)** Heatmap of shared marker expression across major cell types. **(C)** Heatmaps of differentially expressed genes (DEGs) in monocytes, NK, CD4+, CD8+ T cells, and Tregs. **(D)** Bar plot of proportions of key subsets within NK, CD4+, CD8+, and monocyte compartments. **(E)** Violin plots of marker expression across severity groups. **(F)** Volcano plots and violin plots of significant DEGs across disease groups (*adj. p* < 0.05, log2FC > 0.5). UMAP, Uniform Manifold Approximation and Projection; scRNA-seq, single-cell RNA sequencing; scATAC-seq, Assay for Transposase-Accessible Chromatin with high-throughput sequencing; gdT, Gamma Delta T cells; Treg, Regulatory T cells; MAIT, Mucosal-Associated Invariant T cells; dnT, Double Negative T cells; TCM, T Central Memory cells; TEM, T Effector Memory cells; NK, Natural Killer cells; MONO, Monocytes; cDC2, Conventional Dendritic Cells type 2; pDC, Plasmacytoid Dendritic Cells; HSPC, Hematopoietic Stem and Progenitor Cells. Statistical analysis is denoted by asterisks: ****p < 0.0001, ***p < 0.001, **p < 0.01, *p < 0.05, and “ns” non-significant difference.

Subsequent unsupervised clustering (**Figure 1D**) revealed four distinct subclusters within the monocyte population: classical *CD14* ^+^ *CD16* ^-^ monocytes, proinflammatory *CD14* ^+^ *CD16* ^-^ monocytes, intermediate *CD14* ^+^ *CD16* ^+^ monocytes, and non-classical *CD14* ^-^ *CD16* ^+^ monocytes (**Figures 1D**, **2A, D**). Additionally, the *CD4* ^+^ T cell compartment included two subclusters of T central memory cells (TCM) 1 and 2, and a mixed cluster of naïve and TCM *CD4* ^+^ T cells (*CD4* ^+^ naïve/TCM). The *CD8* ^+^ T cell population had distinct subclusters: naïve *CD8* ^+^ T cells, a combined TCM/TEM population, and subclusters 1, 2, and 5 of *CD8* ^+^ T effector memory (TEM) cells. NK cells were further categorized into NK *CD56* ^bright^ cells and subclusters 1, 2, and 5 of NK *CD56^dim^* cells.

Most cell subtypes did not show differential patterns when comparing controls and different severities of AA. However, we observed a significant reduction in *CD14* ^+^ *CD16* ^-^ monocytes in both mild and severe AA (p < 0.001) (**Figure 1F**). Additionally, mild AA showed increased *CD4* ^+^ TCM (p < 0.05) and NK *CD56* ^dim^ cells (p < 0.05), while severe AA had an elevated subpopulation of *CD8* ^+^ TEM cells (p < 0.05). No significant differences were found in other cellular subtypes between AA patients and healthy subjects.

### AA patients display increased transcriptional activity across circulating immune subsets

All patients with AA showed an overall increase in marker number, expression changes, and variability in cell percentages across all PBMC types and subtypes (**Figure 2E**). These effects were more pronounced in severe cases, with monocytes being most affected, followed by NK and *CD8* ^+^ T cells.

Specifically, *CD14* ^+^ proinflammatory and *CD16* ^+^ monocytes had the highest number of altered markers. Significant changes were also seen in NK cells, *CD8* ^+^ T cells, pDCs, and cDC2s, highlighting a dysregulated innate immune response (**Figure 2E**). To a lesser extent, increases were observed in adaptive immune cells like HSPCs, plasmablasts, and B cell subtypes. Tregs showed minimal changes, suggesting a possible failure to control inflammatory activity.

### Lineage-specific transcriptional signatures highlight functional skewing in monocytes, NK cells, and T cells

Single-cell transcriptomic analysis identified lineage-specific transcriptional profiles across key populations in PBMCs from patients with AA, revealing distinct functional states in monocytes, NK cells, and T cell subsets (**Figure 2C**).

In summary, monocyte subsets in AA exhibit distinct yet overlapping transcriptional profiles. Shared expression of *LYZ*, *FCN1*, *IRAK3*, and *CD83* across all subsets reflects a conserved monocytic core program involved in innate immune sensing and regulation. Transcription factors such as *ZEB2* and *NFKBIZ* were also broadly expressed, supporting a shared inflammatory potential. *CD14* ^+^ monocytes showed enrichment in matrix remodeling (*VCAN*) and inflammatory metabolism (*NAMPT*, *PLTP*, *PLIN2*), while *CD16* ^+^ monocytes upregulated pro-inflammatory alarmins (*S100A9*) and cytokines (*IL1B*), stress-responsive transcription factors (*FOSB*, *BACH1*), and effector molecules like *TNFAIP2*. *CD14* ^+^/*CD16* ^+^ monocytes displayed a hybrid profile overlapping with both classical and non-classical subsets.

NK cell subpopulations displayed distinct transcriptional signatures. NK *CD56* ^bright^ cells expressed higher levels of *XCL1*, *XCL2*, *IL7R*, and *KLRF1*, suggesting immunoregulatory and chemotactic roles. In contrast, NK *CD56* ^dim^ cell subsets were enriched for cytotoxic mediators including *PRF1*, *GZMB*, *GNLY*, *TYROBP*, and *NKG7*, consistent with their established effector function.

*CD4* ^+^ T cells exhibited gene signatures associated with memory and survival (*CD27*, *ICOS*, *BCL2*), along with markers indicative of chemotactic activity and tissue remodeling (*CCL5*, *ADAM8*). *CD8* ^+^ T cells showed strong expression of cytotoxic mediators (*GZMA*, *GZMH*, *GZMK*) and proliferation-associated genes (*MKI67*, *TOP2A*), indicating active effector function and clonal expansion.

*CD4* ^+^ and *CD8* ^+^ TCM displayed transcriptional signatures consistent with a resting memory state and potential for tissue interaction, marked by *CD27*, *ICOS*, *BCL2*, *ADAM8*, *STMN1*, and *CCL5*. In contrast to *CD8* ^+^ TEM, they lacked prominent effector and proliferative markers such as *GZMH*, *MKI67*, *TOP2A*, and *TIGIT*, supporting their identity as less activated, long-lived memory T cells.

### Immune cell activation and effector programs are amplified in severe versus mild AA

Gene expression analysis revealed a markedly higher transcriptional activity in severe AA patients compared to those with mild disease and healthy controls (**Figures 2E–G**). This increased activity was observed across nearly all cell subsets, with the most prominent changes in *CD14* ^+^ and *CD14* ^+^ *CD16* ^+^ monocytes, NK *CD56* ^bright^ cells, *CD4* ^+^ naïve/TCM, and *CD8* ^+^ TCM/TEM populations, reflecting a broad and coordinated response to severe activation. In contrast, mild AA cases displayed a more heterogeneous pattern, with modest activation in *CD14* ^+^ *CD16* ^+^ monocytes and NK *CD56* ^dim^ 1 cells, and potential regulatory engagement by Tregs suggesting early-stage inflammatory modulation.

In severe AA, upregulated genes were enriched in pathways related to MHC class I presentation, chemokine signaling, damage-associated molecular patterns (DAMPs), cell migration, and antimicrobial responses (**Figure 2G**). Notably, NAMPT, FOS, S100A9, PRF1, and GNLY were strongly upregulated in monocytes, NK cells, and *CD8* ^+^ TEM, alongside increased expression of HLA-C and B2M, indicative of heightened antigen presentation and cytotoxic activity (**Figure 2B**). In contrast, mild AA showed higher expression of IL7R, TSC22D3, DUSP6, and ZFP36L2, suggesting preserved homeostatic regulation and anti-inflammatory signaling, particularly in memory T cells and monocytes.

Additionally, key transcription factors such as *FOS*, *JUNB*, and *NR4A3*, along with the inflammation modulator *ZFP36*, were significantly overexpressed in severe AA, moderately expressed in mild cases, and downregulated in healthy controls, further highlighting the progressive immunopathology associated with disease severity (**Figure 2G**).

### Cytokine and chemokine expression profiles indicate enhanced immune activation and tissue-directed recruitment of peripheral immune cells

**Figure 3:**
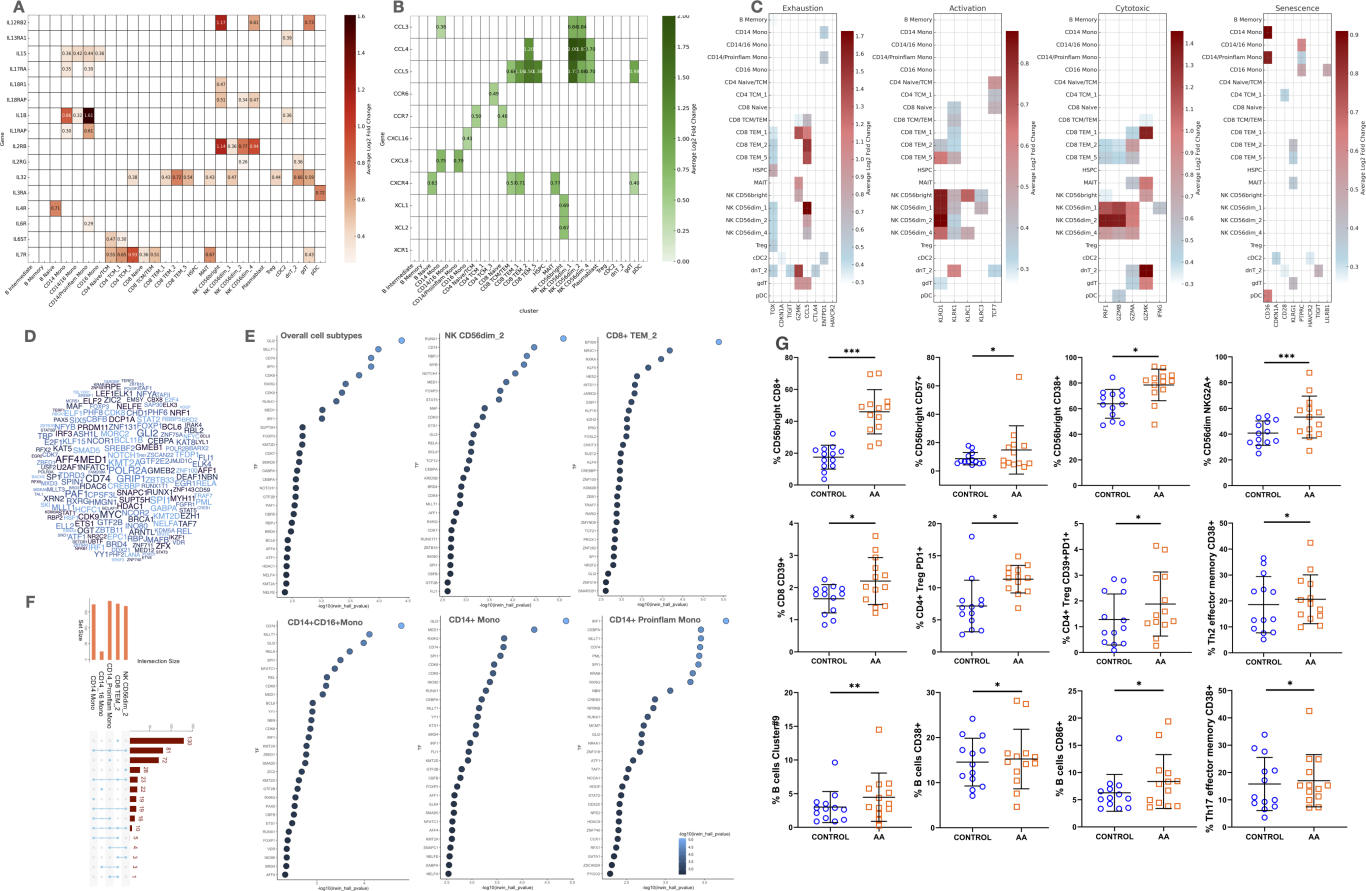
Cytokine and chemokine Expression, cell functionality markers, transcription factor predictions, and comparative analysis in AA. **(A, B)** Heatmaps of cytokine and chemokine expression across cell types. **(C)** Heatmaps of functional signatures: senescence, exhaustion, activation, cytotoxicity. **(D)** Word cloud of top transcription factors (TFs) from ATAC-seq data. **(E)** Dot plots showing top predicted TFs per cell type (NK, CD8+, monocytes). **(F)** Bar plot of pathway overlap among DEGs. **(G)** Flow cytometry validation of immune subsets. Statistical analysis is denoted by asterisks: ***p < 0.001, **p < 0.01, *p < 0.05.

Our integrated single-cell transcriptomic and chromatin accessibility profiling revealed diverse functional programs across PBMC subsets in patients with AA. Distinct expression patterns were observed for cytokine signaling, chemokine-mediated migration, cytotoxicity, exhaustion, and cellular senescence (**Figure 3A–C**).

#### Cytokine and cytokine receptor expression: Th1, Th2, and Th17 axis engagement in AA

Monocytes, particularly CD14^+^ classical and proinflammatory subsets, exhibited strong expression of IL1B, IL15, IL17RA, and IL1RAP, indicating engagement of Th1 and Th17 pathways, as well as activation of the IL-1/IL-6/IL-18 axis (**Figure 3A**). This inflammatory profile was further supported by cDC2s, which expressed IL1B and IL13RA1, suggesting additional responsiveness to Th2-mediated signals.

NK *CD56* ^bright^ cells upregulated IL12RB2, IL18R1, and IL18RAP, which are central to Th1-type activation and IFN-γ production, promoting cytotoxic function and inflammatory amplification. Meanwhile, CD56^dim^ subsets expressed *IL32*, *IL2RB*, and in some cases *IL2RG*, indicating responsiveness to γ-chain cytokines and contribution to both effector and homeostatic signaling.

In T cells, expression of IL7R, IL2RG, and IL32 in *CD4* ^+^ and *CD8* ^+^ subsets supports a transcriptional program associated with survival, memory maintenance, and cytokine-driven activation. The presence of *IL4R* and *IL13RA1* in T and B cells, albeit modest, points to partial activation of the Th2 axis, while *IL17RA* expression in monocytes and innate-like lymphocytes confirms a parallel Th17 component.

#### Chemokine and receptor expression: migration, immune polarization, and tissue recirculation

Distinct chemokine and receptor profiles across cells subsets in AA highlight coordinated mechanisms of cell recruitment, tissue homing, and inflammatory amplification (**Figure 3B**). *CD14* ^+^ monocytes and *CD56* ^dim^ NK cells prominently expressed *CCL3*, while *CCL4* and *CCL5* were shared by cytotoxic *CD8* ^+^ TEM cells and γδ T cells, indicating a chemotactic axis involved in mobilizing effector cells to inflammatory sites. Expression of *CXCL8* in *CD14* ^+^ monocytes and *CXCL16* in *CD16* ^+^ monocytes suggests a sequential role in initiating and sustaining inflammation, respectively.

*CXCR4*, a key regulator of cell trafficking, was broadly expressed in B naïve, *CD8* ^+^ TCM/TEM, γδ T cells, and MAIT cells, pointing to a shared mechanism for recirculation and tissue migration. Additional receptors such as CXCR6 and CCR6, found in CD4^+^ memory T cells and innate-like populations, support mucosal and peripheral tissue homing capacities. CCR7 expression in naïve CD4^+^ and CD8^+^ T cells further reflects their readiness for lymph node recirculation and antigen surveillance. Of note, NK *CD56^bright^* cells expressed *XCL1*, a chemokine involved in dendritic cell recruitment and inflammatory modulation, reinforcing their role in orchestrating early immune responses.

### Immune dysfunction signatures define cytotoxic, exhausted, and senescent states in circulating immune cells of AA patients

Our study found significant overexpression of exhaustion markers (*TOX*, *TIGIT*, *GZMK*, *CTLA4*) in DNT 2, indicating chronic antigen stimulation and cell exhaustion in patients with AA (**Figure 3C**). Other cells showed lower levels of these markers, including γδ T cells (*GZMK*), cDC2s (*CDKN1A*, *ENTPD1*, *HAVCR2*), and *CD14^+^* monocytes (*CDKN1A*). Apoptotic markers were limited, with *CASP3* in plasmablasts and *CASP1*/*ANXA5* in monocytes and cDC2s, suggesting higher apoptosis in these cells. In contrast, the anti-apoptotic marker *BCL2* was prevalent in *CD4^+^* T cells, naïve *CD8^+^* T cells, and Tregs, indicating preserved T cell viability. Senescence markers were less common but showed increased susceptibility in monocytes, dendritic cells, and DNT 2 cells in AA patients.

### Distinct transcription factor activity profiles reveal cell type–specific regulatory alterations in circulating immune cells in AA

To explore the regulatory mechanisms driving transcriptional changes in AA, we performed TF enrichment analysis on DEGs from AA patients versus healthy controls (**Figures 3D–F**). The global view (**Figure 3D**) revealed a distributed regulatory landscape, with no single TF dominating across all cell subtypes. This suggests that transcriptional regulation in AA is context-dependent and shaped by cell type–specific TF combinations.

Among all subsets, *CD8* ^+^ TEM 2 cells displayed the highest number of uniquely enriched TFs, indicative of a specialized program linked to cytotoxic function and partial exhaustion. Key TFs such as *TBX21*, *EOMES*, *PRDM1*, and *ZEB2* were selectively enriched, aligning with a memory-effector phenotype.

In contrast, most other TFs were enriched in *CD14* ^+^ monocytes, either exclusively or shared with NK *CD56* ^dim^ 2 cells, reflecting innate regulatory overlap. *CD14* ^+^ proinflammatory monocytes showed enrichment of TFs like IRF1, CEBPA, SPI1, MLLT1, and CD74, associated with interferon signaling, monocyte activation, inflammation, and antigen presentation. Conversely, enrichment of regulatory TFs such as *RXRG* and *KRAB* suggests compensatory anti-inflammatory mechanisms.

NK *CD56* ^dim^ 2 cells expressed TFs related to cytotoxicity and maturation, including *RUNX1*, *STAT5A*, and *NOTCH1*, supporting their effector role. The presence of *FOXP3* suggests activation-modulated state, while *CD74* and MED1 point to enhanced transcriptional activity and responsiveness. This profile partially overlaps with that of *CD8* ^+^ TEM 2 cells, indicating shared effector regulatory pathways.

Several TFs, including *GLI2* (modulator of inflammation via Hedgehog signaling), *MALT1* (NF-κB activator in T and B cells), *FOXP1* (regulator of memory T and B cell differentiation), *RELA* (core NF-κB subunit promoting inflammatory gene expression), and *SPI1* (key driver of monocyte/macrophage identity), were enriched across multiple lineages, underscoring their cross-lineage relevance in AA pathogenesis.

Altogether, these results reveal a complex yet structured regulatory network in AA, driven by both shared and cell type–specific TFs, and highlight candidate master regulators of disease-related immune-related responses.

### Flow cytometry validates altered frequencies of circulating immune subsets in an independent AA cohort

To validate and complement transcriptional insights, we analyzed the frequencies of cell subsets by conventional flow cytometry. Several phenotypically defined populations showed significant differences between AA patients and healthy controls (**Figure 3F**).

Multiple *CD56* ^bright^ NK cell subsets, including *CD8* ^+^, *CD57* ^+^, and *CD38* ^+^ phenotypes, were significantly expanded in AA (p < 0.05), consistent with increased cytotoxic activity, activation and terminal differentiation. The *CD56* ^dim^ *NKGA2A* ^+^ NK subset, involved in cytolytic and regulatory functions, was also significantly increased (p < 0.001).

*CD8* ^+^ T cells expressing *CD39* ^+^, a marker of activation and partial exhaustion, were more abundant in AA (*p < 0.05*). *CD4* ^+^ Tregs showed a significant increase in *CD39* ^+^/PD1^+^ subsets (*p < 0.05*), suggesting an expanded yet potentially dysfunctional suppressive compartment.

Among *CD4* ^+^ T helper subsets, Th2 and Th17 effector memory cells expressing *CD38*+ were significantly elevated (*p < 0.05*), supporting a broader activation state and possible shift toward pathogenic Th polarization.

B cell populations were also altered. B cells expressing *CD38* ^+^ or *CD86* ^+^ were significantly increased in AA (p < 0.01 and *p < 0.05*, respectively), suggesting enhanced activation and antigen-presenting potential.

### Pseudotime analysis reveals skewed differentiation trajectories in circulating immune cells

**Figure 4:**
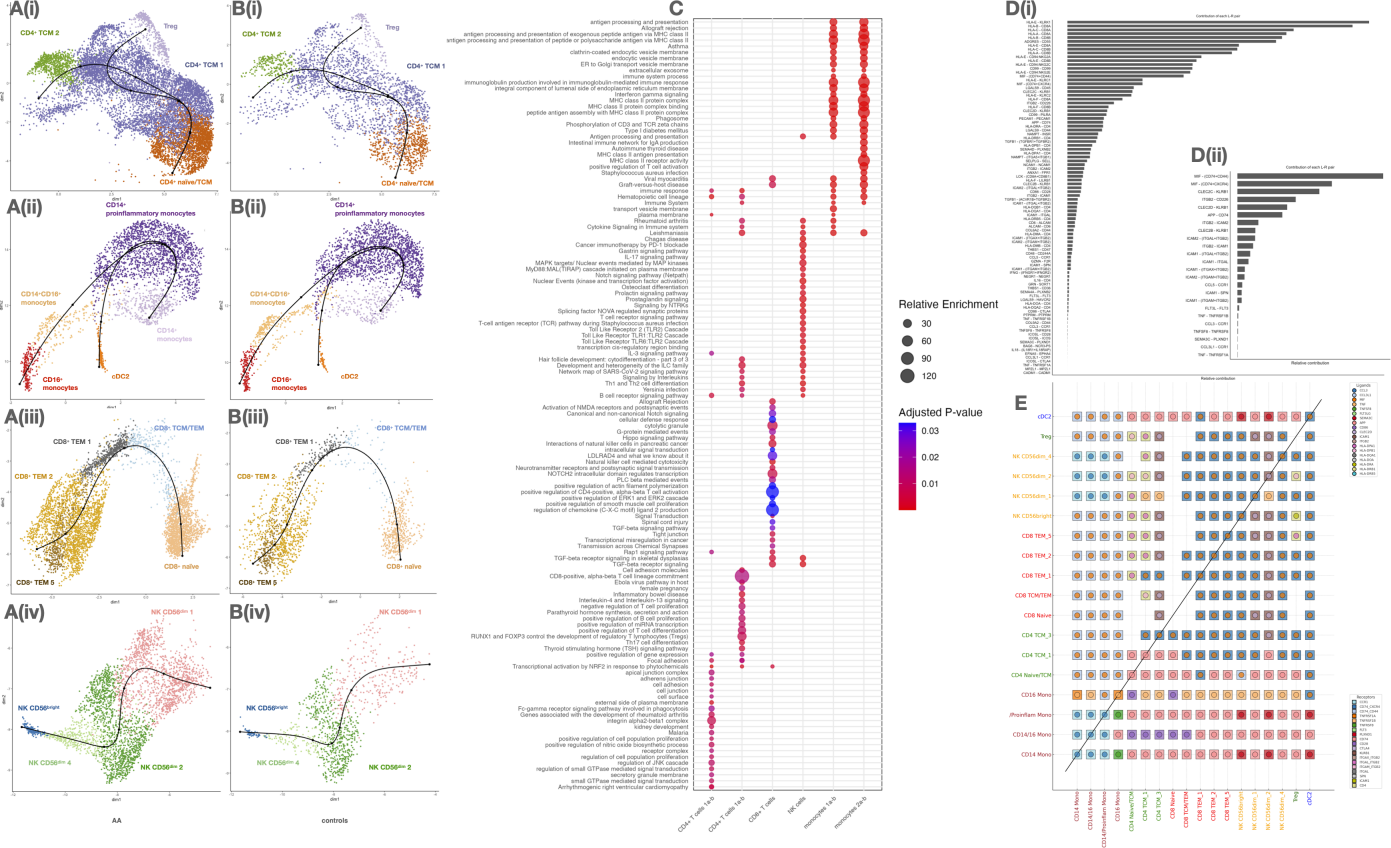
Analysis of immune cell subtypes, functional pathway enrichment, and ligand-receptor interaction preferences in circulating cell subsets of alopecia areata (AA) and control samples. **(A, B)** UMAP plots of major immune subtypes in AA **(A)** and controls **(B)**. **(C)** Dot plot of enriched pathways per cell subtype (size: gene ratio, color: adjusted *p*). **(D)** Receptor–ligand interaction frequency: total (i) and excluding HLA (ii). **(E)** Heatmap of inferred ligand-receptor interactions between immune subsets. Diagonal separates AA vs control comparisons.

To investigate the developmental dynamics, we performed pseudotime trajectory analysis on *CD4* ^+^ T cells, *CD8* ^+^ T cells, monocytes, and NK cells, comparing patients with AA (**Figure 4A**) to healthy controls (**Figure 4B**). These analyses revealed marked shifts in differentiation paths and cell state distributions across cell subsets in AA.

In *CD4* ^+^ T cells (**Figure 4A**(i)), trajectories originated from a *CD4* ^+^ naïve/TCM cluster and diverged toward two distinct fates: Tregs and *CD4* ^+^ TCM 1/2. In AA patients, the *CD4* ^+^ naïve/TCM compartment appeared depleted, while Treg and TCM 2 populations were expanded, suggesting accelerated differentiation or loss of naïve precursors. In contrast, controls (**Figure 4B**(i)) retained a more prominent naïve cluster and balanced distribution along both fates.

Monocytes in AA (**Figure 4A**(ii)) transitioned from *CD14* ^+^ monocytes toward two terminal phenotypes: proinflammatory *CD14* ^+^ monocytes and *CD16* ^+^ monocytes, with an intermediate *CD14* ^+^ *CD16* ^+^ state. The trajectory was markedly skewed towards the proinflammatory trajectory in AA, while in controls (**Figure 4B**(ii)), trajectories remained centered around homeostatic *CD14* ^+^ cells, with more restricted expansion toward inflammatory states.

For *CD8* ^+^ T cells (**Figure 4A**(iii)), the trajectory started from a naïve population and progressed toward *CD8* ^+^ TCM/TEM, branching into two TEM subtypes (TEM 1 and 2) and a terminal TEM 5 subset. The TEM 5 population, enlarged in AA, represents a potentially exhausted or chronically activated effector state. In healthy controls (**Figure 4B**(iii)), differentiation was more constrained, with fewer cells populating terminal effector branches.

NK cell trajectories (**Figure 4A**(iv)) revealed a progression from CD56^bright^ toward multiple CD56^dim^ subsets, notably NK CD56^dim^ 1, which was expanded in AA and occupied the terminal state. This subset may reflect heightened cytotoxic activation or tissue-migratory potential. In controls (**Figure 4B**(iv)), CD56^bright^ cells remained more abundant and less differentiated, suggesting a more regulated NK maturation process.

### Cell–cell communication inference reveals enhanced crosstalk and effector signaling in AA

To investigate systemic dysregulation in AA, we inferred ligand–receptor-mediated communication networks between cell subtypes using a curated interaction model. A global analysis of the aggregated communication landscape revealed a substantial increase in both the number and strength of interactions in AA patients compared to controls, particularly in severe cases (**Supplementary Figure S10**; **Figures 4C–E**). Notably, *CD4* ^+^ TCM, *CD8* ^+^ TEM, CD56^bright^ NK cells, and proinflammatory *CD14* ^+^ monocytes emerged as major hubs of outgoing signaling activity. These findings are consistent with their elevated transcriptional activity and chromatin accessibility previously identified in our dataset.

Further analysis of directional communication patterns confirmed that these subsets act as key signal “senders”, exerting broad influence over other cell subtypes, including Tregs and cDC2 (**Supplementary Figure S11**). At the pathway level, we identified significant contributions to several canonical axes, including MHC class I/II, *CCL*, *CXCL*, *IFN*, and *TNF* signaling (**Supplementary Figure S12**), all of which have been previously implicated in the disruption of IP at the hair follicle level.

Within these networks, monocyte and NK subsets were dominant producers of TNF and type I/II interferon signals, while Tregs and naïve T cells showed markedly lower participation in proinflammatory pathways. This imbalance supports the hypothesis of diminished immunoregulation coupled with enhanced effector signaling in patients with severe AA.

We identified several key ligand–receptor signaling interactions mediating cell-cell crosstalk, including *CLEC2D*–*KLRB1*, facilitating communication between *CD4* ^+^ TCM and NK cells; *HLA-DPB1*–*DPA1*, bridging *CD4* ^+^, *CD8* ^+^, Tregs, and NK subsets; and classic T cell co-stimulatory pairs, such as *LCK*–*CD8* and *CD86*–*CD28*, which are essential for T cell activation and priming.

Cell-cell signals promoting adhesion and migration were mediated by axes like collagen–*CD44*, *PECAM1*–*PECAM1*, *CCL5*–*CCR1*, and *IL16*–*CD4*, guiding cells to inflammatory niches. Additionally, galectin-mediated regulation involving *LGALS1*, *LGALS2*, *LGALS3*, and *LGALS9* emerged as a potential mechanism modulating cell–cell contact, activation, and apoptosis.

We also observed key immunoregulatory interactions, such as *THBS1*–*CD47*/*CD36*, *TGFB1*–*TGFBR1*/*2*, and negative feedback loops through *ANXA1*–*FPR1*, *HLA-F*–*LILRB1*, and *LGALS9*–*CD44*, which may be essential for resolving inflammation and restoring homeostasis.

### Chromatin accessibility profiling identifies epigenetic changes in key circulating immune subsets

Our analysis identified 42,248 significant ATAC peaks across samples, indicating broad chromatin accessibility changes in AA patients. While most cell types showed few changes (**Supplementary Figure S7**), NK cells and *CD8^+^* TCM/TEM had the most peaks. Intermediate B cells and *CD14^+^* monocytes showed more variability, suggesting diverse chromatin dynamics. Increased chromatin accessibility was observed in *CD14^+^* monocytes, NK *CD56^bright^* cells, HSPCs, B cells, plasmablasts, and MAIT cells (**Supplementary Figure S8**). In contrast, naïve T cells, Tregs, and γδ T cells showed fewer changes, indicating stable chromatin regulation.

We identified 261 differentially expressed genes with adjacent open chromatin regions in AA patients (**Supplementary Table S3**). These genes exhibited coordinated regulation at both the epigenetic and transcriptional levels, implicating functional relevance in disease pathology.

In B cells (naïve, intermediate, memory), *GNG7*, *LYN*, and *PAX5* showed the highest number of open chromatin regions, reflecting altered B cell signaling. Among *CD8* ^+^ memory/effector T cells and MAIT cells, *CXCR4*, *ZFP36L2*, and *ZBTB16* stood out—*CXCR4* being a key player in Th1 polarization and lymphocyte trafficking. NK CD56^bright^ cells displayed accessibility at *AUTS2*, IL2RB, and *PLCB1*; notably, *IL2RB* is involved in Th1 and Th2 cytokine signaling via the JAK/STAT pathway. In monocytes, genes such as *NEAT1*, *EVI5*, and *PTPN6* were accessible, the latter regulating JAK/STAT activation. In plasmacytoid dendritic cells, open chromatin at *FCHSD2*, *RNF149*, *IRF7* and *PTPRE* may underpin interferon-related responses. Additionally, *IL4R*, enriched in B naïve cells, links to Th2 signaling. These findings suggest epigenetic activation of pathways including Th1, Th2, interferon-JAK/STAT, and B cell signaling across distinct subsets in AA.

### Gene module analysis highlights coordinated regulatory programs in circulating immune cells

**Figure 5:**
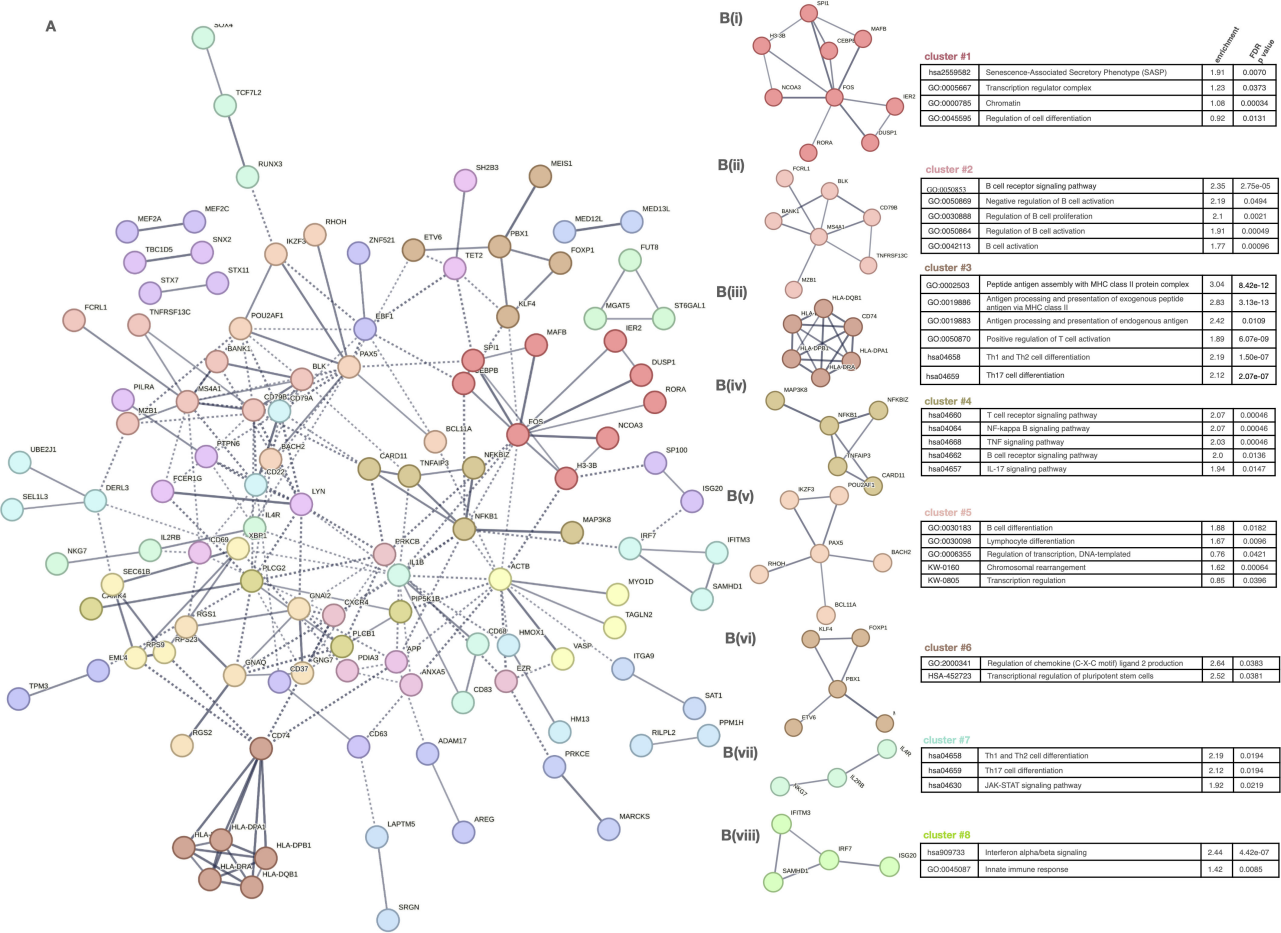
Network analysis of gene clusters and functional pathway enrichment in immune cell subtypes. **(A)** Network plot of gene-gene interactions, colored by cluster. **(B) (i–viii)** Detailed cluster-specific pathway enrichment: • C1: Nucleosome, chromatin, senescence • C2: B cell activation • C3: Antigen processing, T cell differentiation • C4: Inflammatory signaling (NF-κB, TNF, IL-17) • C5: Transcriptional control, lymphocyte differentiation • C6: Chromatin modifiers • C7: Th1/Th17 differentiation, JAK–STAT • C8: IFN signaling, innate immunity.

To explore their functional organization, we constructed a protein–protein interaction (PPI) network using STRING, comprising 259 nodes and 226 edges, significantly above random expectation (p < 1.0e-16) (**Figure 5A**). This network revealed a highly interconnected regulatory structure, indicative of biologically meaningful co-regulation among these genes.

To further dissect the biological functions of this regulatory network, we applied unsupervised clustering, resulting in eight distinct gene modules (**Figure 5B**(i-viii)). Each cluster was functionally annotated via enrichment analysis (Reactome, GO, KEGG), revealing discrete activation and regulatory pathways relevant to AA pathogenesis.

Clusters 1 and 5 captured modules involved in transcriptional control, chromatin dynamics, and lymphocyte differentiation, indicating a general dysregulation of gene regulatory programs in adaptive immune cells. These included transcriptional regulators such as *ETV6*, *NCOR2*, *MAFB*, *MEF2C*, *TCF3*, and *SPIB*, which may modulate key pathways of cell activation and maturation, contributing to the altered functional states observed in AA.

Centered on antigen presentation and effector T cell responses, clusters 3 and 4 were enriched for MHC class I and II pathways and T cell activation genes (e.g. *HLA-DPB1*, *CD74*), as well as TNF, IL-17, and TCR signaling components (e.g. *TNFAIP3*, *BCL3*), respectively.

Clusters 6 and 7 were associated with broader modulation, encompassing chemokine signaling and T helper cell polarization (Th1, Th2, Th17) via the JAK–STAT pathway, highlighting disrupted differentiation programs and increased plasticity.

Finally, cluster 8 represented a coherent interferon response module, including type I IFN effectors, consistent with the systemic antiviral-like signature previously reported in AA.

## Discussion

Our study provides the first comprehensive multi-omic characterization of peripheral immune cells in AA, integrating single-cell RNA sequencing and chromatin accessibility profiling. This strategy enables high-resolution delineation of transcriptional states, regulatory circuits, and upstream drivers of immune dysregulation, allowing in-depth comparisons across disease severities.

We identified proinflammatory monocytes, *CD8* ^+^ effector memory T cells, and NK cell subsets as major contributors to systemic activation in AA. These populations exhibited elevated transcriptional activity, increased chromatin accessibility, and enrichment of transcription factors involved in cytotoxicity, antigen presentation, and inflammation. In contrast, regulatory and naïve subsets displayed limited transcriptional and epigenetic changes, indicating a selective activation of effector mechanisms.

Genes related to both innate and adaptive immunity—including memory cell markers and skin-homing/recirculation signatures—were overexpressed in these subsets, highlighting their peripheral readiness to engage in tissue-specific responses. Importantly, these transcriptional profiles mirrored previously characterized lesional features such as *IFN*-stimulated genes, cytotoxic granules (e.g., *GZMB*, *GNLY*), and MHC class I/II upregulation, implicating systemic engagement in the collapse of HF’s IP (24).

Notably, several subsets—including Tregs, DNTs, and monocytes—expressed regulatory molecules alongside exhaustion markers, reflecting a dysfunctional attempt to suppress inflammation. These states were accompanied by Th1-, Th2-, and Th17-associated cytokine programs and cytotoxic and antigen-presenting pathways, all previously implicated in the collapse of HF’s IP in AA (29).

Our findings are consistent with previous reports of perifollicular infiltration by *CD8* ^+^ and NK cells, overexpression of MHC class I and II molecules in follicular keratinocytes, and dysfunction of regulatory T cells in both blood and tissue (24, 63). Importantly, our data now extend these observations by demonstrating that these immunological pathways are also systemically dysregulated, particularly in patients with severe AA (64).

In severe AA, we observed a coordinated activation across transcriptional, epigenomic, and signaling layers within effector subsets—most prominently proinflammatory *CD14* ^+^ monocytes, *CD8* ^+^ TEM cells, and *CD56* ^bright^ NK cells. These populations exhibited increased chromatin accessibility at loci involved in antigen presentation, chemokine signaling, and interferon responses, accompanied by strong upregulation of cytotoxic mediators such as *PRF1*, *GNLY*, and *IFN*-stimulated genes. Importantly, these axes replicate mechanisms observed in lesional HF during IP collapse (24, 29, 64).

In contrast, mild AA was associated with intermediate activation profiles. Cell types such as NK *CD56* ^dim^ cells and *CD14* ^+^ *CD16* ^+^ monocytes showed moderate increases in transcriptional activity, while regulatory subsets like Tregs retained expression of homeostatic and anti-inflammatory genes (e.g., *IL7R*, *TSC22D3*, *ZFP36L2*). Interestingly, some of these cells co-expressed exhaustion markers (e.g., *CTLA4*, *TIGIT*), suggesting that attempts to restrain inflammation may become progressively dysfunctional with increasing disease severity. This may help explain the transition from mild to chronic disease and the associated loss of treatment responsiveness (63).

Moreover, our pseudo-time trajectory analyses revealed a skewing of differentiation pathways in AA toward inflammatory and terminal effector states, particularly in monocytes, *CD8* ^+^ T cells, and NK cells. These shifts were accompanied by the contraction of naïve and precursor compartments, further indicating disrupted homeostasis. Such patterns of accelerated differentiation may underlie disease chronicity and relapse.

Adding another layer of evidence, cell-cell communication analysis uncovered a dense and restructured ligand-receptor interaction network in severe AA. Proinflammatory subsets such as *CD14* ^+^ monocytes, *CD8* ^+^ TEM cells, and *CD56* ^bright^ NK cells were identified as dominant signaling hubs, secreting *TNF*, *IFN*, and chemokine signals and orchestrating recruitment via *CCL5*–*CCR1*, *XCL1*–*XCR1*, and *CXCL10*–*CXCR3* axes. These findings are consistent with cytokine elevation profiles previously described in the serum and skin of AA patients (65–67).

Crucially, all these levels of analysis were consistent in identifying effector populations as key drivers of dysregulation, particularly in severe AA. Conversely, preserving regulatory and anti-inflammatory programs in mild cases suggests a window of immune plasticity that may allow for disease reversal before the complete collapse of regulatory control. This concept aligns with clinical observations of spontaneous remission or effective treatment response in early AA and resistance or chronicity in more advanced stages (5).

Previous studies have examined this activation in limited cell subtypes through cytometry, or indirectly via transcriptomic analysis or measuring proteins like cytokines in bulk samples (66–72). Our findings are consistent with their results showing altered monocytes, *CD4* ^+^ T cells, *CD8* ^+^ T cell, and NK cells dysregulation (73–75), transcriptomic changes (76), cytokine/chemokine activation (24, 77–80), epigenetic changes (35), immunotolerance (81, 82), and impaired regulatory T cell function (83). Despite variations in specific cell subtypes and cytokines involved, findings are consistent, suggesting circulating immune cells critically contribute to AA’s development and progression, with implications for targeted therapies.

Notably, our data reveal that systemic immune dysregulation is evident even in patients with mild AA. This raises important clinical implications regarding early therapeutic intervention. Although JAK inhibitors (JAKi) such as baricitinib, ritlecitinib, and deuruxolitinib have been approved for the treatment of severe AA, their potential benefits in earlier disease stages remain to be fully explored (84). Identifying predictors of progression could allow for timely stratification and personalized management of AA patients, an area that warrants prospective investigation.

These findings align with emerging clinical trial evidence suggesting that early intervention with JAK inhibitors may improve long-term outcomes by preventing irreversible follicular damage. Stratifying patients based on peripheral immune activation—particularly JAK/STAT-associated transcriptional signatures—could help identify those most likely to benefit from early targeted therapy.

Consistent with these findings, we identified cell type–specific enrichment of TFs such as SPI1 in monocytes (85), PRDM1 (Blimp-1) in *CD8* ^+^ effector T cells (86), and ZEB2 in NK and *CD8* ^+^ memory subsets (87). These TFs are known regulators of monocyte activation, cytotoxic T cell differentiation, and effector memory programming, respectively. Although our study inferred TF activity using integrative scRNA-seq and scATAC-seq analyses, experimental validation is needed to confirm their functional roles in AA. Future studies could employ CRISPR interference or activation (CRISPRi/a), overexpression or knockdown in ex vivo PBMCs, and ChIP-seq to elucidate TF-target relationships and regulatory networks. These approaches may clarify whether such TFs contribute directly to the dysregulated immune circuits observed in AA and support their potential as biomarkers or therapeutic targets.

### Discrepancies, strengths, and limitations

Our study benefits from high-quality data and robust multi-omic integration, enabling confident identification of programs across cell types and disease severities. The confirmation of severity-associated signatures and their validation in an independent cohort via flow cytometry further reinforces our findings’ reproducibility and translational relevance.

One limitation of our study is the absence of significant enrichment for *CD8* ^+^ T cells in peripheral blood, which contrasts with some earlier reports. This discrepancy may reflect compartmentalization of pathogenic subsets within lesional skin, rather than circulation. In addition, mast cells and tissue-resident immune cells—which play key roles in local IP collapse—were not evaluated in this study. Another constraint is the relatively modest sample size; however, this is partially offset by the high dimensionality and resolution of the single-cell data, as well as the validation of key findings through independent flow cytometry analyses. Future studies involving larger cohorts will be important to confirm and expand upon these results.

Although the full study cohort was matched for age, sex, and ethnicity, the subset selected for single-cell profiling showed some imbalance in sex distribution due to differences in sample quality and sequencing performance. To reduce potential confounding, all statistical models were adjusted for sex, age, and SALT score. Nonetheless, this imbalance represents a potential limitation when interpreting sex-sensitive transcriptional differences at the cell-type level.

Finally, because the definition of the “severe” AA group was based on both disease extent (≥50% scalp involvement) and chronicity (≥1 year duration), we cannot fully separate the relative contributions of disease severity and duration to the observed immune dysregulation. It is likely that both factors interact and contribute to the progressive immunopathology identified.

In summary, our study reveals distinct transcriptional and regulatory alterations in circulating immune cells of AA patients, with progressive changes correlating with disease severity. These findings expand current knowledge of AA pathobiology and provide a systemic perspective on immune activation that complements previous skin-focused studies.

## Conclusion

This study presents the first integrated single-cell transcriptomic and epigenetic map of circulating immune cells in AA, revealing severity-dependent immune activation and regulatory imbalance. The identification of specific dysregulated pathways and immune cell subsets provides valuable insight into the systemic nature of AA as an inflammatory disease. Notably, even patients with mild disease display molecular evidence of systemic immune activation, challenging the notion that AA is solely a localized scalp disorder. These findings pave the way for biomarker discovery and support the development of stage-specific, targeted immunomodulatory therapies tailored to individual immune profiles.

Our findings suggest that systemic immune activation is already present in patients with mild AA, raising important questions about the timing and intensity of therapeutic intervention. Identifying molecular or cellular predictors of progression from mild to severe disease could enable early stratification of patients who may benefit from systemic treatments. Currently, JAK inhibitors such as baricitinib and ritlecitinib are approved primarily for severe cases of AA; however, the observed activation of IFN- and JAK/STAT-related pathways in mild disease suggests that early targeting of these pathways may be beneficial in preventing disease escalation. Prospective studies are needed to determine which immunological features best predict progression and could guide early therapeutic decisions.

## Data Availability

The datasets presented in this study can be found in online repositories. The names of the repository/repositories and accession number(s) can be found below: https://www.ncbi.nlm.nih.gov/geo/query/acc.cgi?acc=GSE277469, GSE277469.
