# Supplementary material for: Integrated single-cell chromatin and transcriptomic analyses of peripheral immune cells in patients with alopecia areata
Source: Front Immunol. 2025 Jul 2;16:1565241. doi: 10.3389/fimmu.2025.1565241 (PMC12263667; doi:10.3389/fimmu.2025.1565241)
Supplement: Supplementary file 1 [file Table1.docx]

**Supplementary Information**

**Integrated single-cell chromatin and transcriptomic analyses of peripheral immune cells in patients with alopecia areata**

**Authors**: ^1,*^ Jesus Gay-Mimbrera, ^1,3, *^Pedro J Gómez-Arias, ^2^ Pablo Álvarez-Heredia, ^2^Alexander Batista, ^3^Irene Rivera-Ruiz, ^1^Macarena Aguilar-Luque,  ^1,3^ Miguel Juan-Cencerrado, ^1,3^ Carmen Mochón-Jiménez, ^1^Álvaro Cebrián-García, ^4^ Eloísa Andújar Pulido, ^4^ Mónica Pérez-Alegre, ^2,5,†^ Alejandra Pera, ^1,2,6,†^ Juan Ruano

^1^ Inflammatory Immune-Mediated Chronic Skin Diseases Laboratory (GC26), Maimonides Biomedical Research Institute of Cordoba (IMIBIC)/University of Cordoba/Reina Sofia University Hospital, Menendez Pidal Ave, 14004, Córdoba, Spain.

^2^Immunology and Allergy Group (GC01), Maimonides Biomedical Research Institute of Cordoba (IMIBIC)/University of Cordoba/Reina Sofia University Hospital, Menendez Pidal Ave, s/n, 14004 Cordoba, Spain.

^3^ Department of Dermatology, Reina Sofía University Hospital, Menéndez Pidal Ave, 14004, Córdoba, Spain.

^4^ Genomic Unit, Andalusian Molecular Biology and Regenerative Medicine Center (CABIMER), CSIC-University of Seville-University Pablo de Olavide, Avda. Americo Vespucio 24. Edif. CABIMER Parque Científico y Tecnológico Cartuja, 41092, Seville, Spain.

^5^Department of Cell Biology, Physiology and Immunology, University of Cordoba, Av. Menendez Pidal s/n, 14004 Cordoba, Spain.

^6^Department of Medicine, University of Córdoba, Menendez Pidal Ave, 14004, Córdoba, Spain.

**Supplementary Tables**

**Table S1. Markers of exhaustion, senescence or apoptosis of immune cells.**

| **Marker Symbol** | **Features** | **Cells Found As Exhaustion Marker** |
| --- | --- | --- |
| **Exhaustion markers** | | |
| PDCD1 | Programmed cell death protein 1 | CD8+ T cells, TILs |
| LAG3 | Lymphocyte-activation gene 3 | CD8+ T cells, TILs |
| HAVCR2 | T cell immunoglobulin and mucin-domain containing-3 | CD8+ T cells, TILs |
| TIGIT | T cell immunoreceptor with Ig and ITIM domains | CD8+ T cells, TILs |
| CTLA4 | Cytotoxic T-Lymphocyte Associated protein 4 | CD8+ T cells, TILs |
| CD244 | 2B4 | NK cells, CD8+ T cells |
| CD160 | CD160 | T cells, NK cells |
| EOMES | Eomesodermin | CD8+ T cells |
| TOX | Thymocyte selection-associated high mobility group box protein | CD8+ T cells |
| PRDM1 | BLIMP-1 | B cells, CD8+ T cells |
| ENTPD1 | CD39 | T cells, NK cells |
| CCL5 | RANTES | T cells, NK cells |
| CXCL9 | MIG | T cells, NK cells |
| CXCL10 | IP-10 | T cells, NK cells |
| CXCL13 | BLC | B cells, T cells |
| CXCR3 | CXCR3 | T cells, NK cells |
| PDCD1LG2 | PD-L2 | T cells |
| BTLA | B and T lymphocyte attenuator | T cells, B cells |
| CD276 | B7-H3 | T cells, NK cells |
| IDO1 | Indoleamine 2,3-dioxygenase 1 | T cells, NK cells |
| LTA | Lymphotoxin alpha | T cells |
| HIF1A | Hypoxia-inducible factor 1-alpha | T cells |
| CCL22 | MDC | T cells |
| CCL28 | MEC | T cells |
| IL10 | Interleukin-10 | T cells, B cells |
| TGFB1 | Transforming growth factor beta 1 | T cells |
| FOXP3 | Forkhead box P3 | T cells, B cells |
| CCR4 | C-C chemokine receptor type 4 | T cells |
| CCR8 | C-C chemokine receptor type 8 | T cells |
| CD276 | B7-H3 (repeated) | T cells, NK cells |
| VISTA | V-set immunoregulatory receptor | T cells |
| NR4A1 | Nuclear receptor subfamily 4 group A member 1 | T cells |
| NR4A2 | Nuclear receptor subfamily 4 group A member 2 | T cells |
| NR4A3 | Nuclear receptor subfamily 4 group A member 3 | T cells |
| AHNAK | AHNAK nucleoprotein | T cells |

| **Senescence and apototic markers** | | |
| --- | --- | --- |
| CDKN2A | Cyclin-dependent kinase inhibitor 2A | Various aging or stressed cells |
| GLB1 | Senescence-associated β-galactosidase | Senescent cells |
| CDKN1A | Cyclin-dependent kinase inhibitor 1A | Cells undergoing DNA damage response |
| TP53 | Tumor protein p53 | Cells undergoing DNA damage response or stress |
| HMGB1 | High mobility group box 1 | Stressed or damaged cells |
| IL6 | Interleukin 6 | Inflammatory cells |
| IL8 | Interleukin 8 | Inflammatory cells |
| DEC1 | Differentiated embryo chondrocyte expressed gene 1 | Stressed or senescent cells |
| CASP3 | Caspase 3 | Apoptotic cells |
| BAX | BCL2 associated X, apoptosis regulator | Apoptotic cells |
| FAS | Fas cell surface death receptor | Cells undergoing apoptosis |
| CASP8 | Caspase 8 | Cells initiating apoptosis |
| CASP9 | Caspase 9 | Cells in intrinsic apoptosis pathway |
| BCL2 | B-cell lymphoma 2 | Cells evading apoptosis |
| BAD | BCL2 associated agonist of cell death | Apoptotic cells |
| BCL2L11 | BCL2 like 11 | Apoptotic cells |
| BAK | BCL2 antagonist/killer | Apoptotic cells |
| APOPT1 | Apoptogenic-1, mitochondrial cysteine protease | Apoptotic cells |

**Table S2. Panel of Flow Cytometry Markers for T Cell and Monocyte Characterization in Alopecia Areata Studies**

| **Panel** | **Marker** | **Supplier** | **Clone** | **Catalog number** |
| --- | --- | --- | --- | --- |
| **T cells** | CD3 | INVITROGEN | PECY5.5 | 35-0036-42 |
|  | CD4 | BD | BV510 | 562970 |
|  | CD8 | BD | BV605 | 564116 |
|  | CD127 | BD | BV711 | 563165 |
|  | CD25 | BD | PE | 555432 |
|  | CD39 | BD | BV650 | 563681 |
|  | CCR6 | BD | APCR700 | 565173 |
|  | CXCR3 | BIOLEGEND | APC-FIRE750 | 353754 |
|  | CD45RA | BD | PERCPCY5.5 | 563429 |
|  | CD28 | BD | BUV737 | 612815 |
|  | CCR7 | BD | BB515 | 566764 |
|  | CD57 | BD | PECF594 | 562488 |
|  | PD1 | MILTENYI | APC | 130-120-382 |
|  | CD38 | BD | PECY7 | 560677 |
|  | L/D | THERMOFISHER | UV1 | L23105 |
| **Monocytes** | CD38 | BD | PECY7 | 560677 |
|  | L/D | THERMOFISHER | UV1 | L23105 |
|  | CD3 | INVITROGEN | PECY5.5 | 35-0036-42 |
|  | CD11c | BIOLEGEND | PEDAZZLE594 | 337228 |
|  | HLADR | BD | BV510 | 563083 |
|  | CX3CR1 | BD | BB700 | 746135 |
|  | CD14 | MILTENYI | APCVIO770 | 130-113-144 |
|  | NKG2D | BD | APC | 558071 |
|  | CD86 | BD | BV711 | 563158 |
|  | NKG2A | MILTENYI | PE | 130-113-566 |
|  | CD56 | BD | APC-R700 | 565139 |
|  | CD16 | BD | BV786 | 563690 |
|  | CD19 | BD | BV650 | 563227 |
|  | CD62L | BD | BB515 | 565037 |
|  | CD57 | BD | PECF594 | 562488 |
|  | CD8 | BD | BV605 | 564116 |

**Table S3. List of 261 differentially expressed genes in AA compared to controls identified by scRNAseq with proximal significantly open chromatin regions detected by scATACseq.**

|  |  |  |  | **scATAC-seq** | | | **scRNA-seq** | |
| --- | --- | --- | --- | --- | --- | --- | --- | --- |
| **gene** | **definicion** | **cells** | **# cells** | **# ATAC peaks** | **Avg**  **log2FC** | **log10 p val adj** | **Avg log2FC** | **log10 p val adj** |
| CXCR4 | C-X-C motif chemokine receptor 4 | B Naive, MAIT, CD8 TCM/TEM | 3 | 58 | 0.605 | 2.60e-07 | 0.625 | 5.14e-77 |
| AUTS2 | activator of transcription and developmental regulator | NK CD56bright, B Naive, B Intermediate, pDC, HSPC | 5 | 45 | 1.434 | 5.26e-21 | 0.846 | 5.07e-60 |
| HLA-DQA1 | major histocompatibility complex, class II, DQ alpha 1 | B Naive, B Intermediate, B Memory | 3 | 42 | 0.895 | 6.76e-16 | 1.369 | 0.00e+00 |
| PRDM2 | PR/SET domain 2 | B Naive, B Intermediate, B Memory | 3 | 37 | 0.909 | 7.90e-23 | 0.686 | 1.01e-114 |
| LYN | LYN proto-oncogene, Src family tyrosine kinase | B Naive, B Intermediate, B Memory, CD16 Mono, pDC | 5 | 35 | 2.285 | 2.34e-53 | 0.966 | 6.77e-207 |
| GNG7 | G protein subunit gamma 7 | B Naive, B Intermediate, B Memory, Plasmablast | 4 | 35 | 3.579 | 8.36e-26 | 0.691 | 0.00e+00 |
| PAX5 | paired box 5 | B Naive, B Intermediate, B Memory | 3 | 33 | 1.520 | 2.45e-39 | 1.018 | 0.00e+00 |
| TRIO | trio Rho guanine nucleotide exchange factor | B Naive, B Intermediate, B Memory, Plasmablast | 4 | 31 | 1.289 | 1.79e-21 | 0.701 | 4.05e-186 |
| MTSS1 | MTSS I-BAR domain containing 1 | B Naive, B Intermediate, B Memory | 3 | 30 | 1.069 | 1.59e-23 | 0.709 | 2.93e-95 |
| CHPT1 | choline phosphotransferase 1 | B Naive, B Intermediate, B Memory | 3 | 24 | 1.033 | 4.16e-29 | 0.658 | 4.40e-272 |
| NEAT1 | nuclear paraspeckle assembly transcript 1 | CD14 Mono, CD16 Mono | 2 | 24 | 0.942 | 6.67e-33 | 1.354 | 1.85e-216 |
| CD83 | CD83 molecule | B Naive, B Intermediate, B Memory, CD16 Mono | 4 | 23 | 0.579 | 3.48e-10 | 1.238 | 2.97e-294 |
| PLCG2 | phospholipase C gamma 2 | B Naive, B Intermediate, B Memory | 3 | 22 | 0.760 | 5.39e-06 | 0.644 | 2.00e-81 |
| ZBTB16 | zinc finger and BTB domain containing 16 | gdT, MAIT | 2 | 22 | 1.980 | 9.60e-40 | 0.620 | 8.25e-120 |
| FCHSD2 | FCH and double SH3 domains 2 | B Naive, B Intermediate, B Memory, pDC | 4 | 21 | 2.606 | 3.14e-41 | 1.062 | 1.82e-208 |
| CD74 | CD74 molecule | B Naive, B Intermediate, B Memory | 3 | 21 | 0.902 | 1.56e-17 | 2.492 | 0.00e+00 |
| TPD52 | tumor protein D52 | B Naive, B Intermediate, Plasmablast | 3 | 21 | 0.566 | 1.00e+00 | 0.724 | 2.73e-169 |
| IL2RB | interleukin 2 receptor subunit beta | NK CD56bright | 1 | 21 | 1.156 | 4.43e-33 | 1.144 | 2.74e-195 |
| BACH2 | BTB domain and CNC homolog 2 | CD8 Naive, B Naive | 2 | 19 | 0.531 | 3.16e-20 | 0.936 | 0.00e+00 |
| PLCB1 | phospholipase C beta 1 | gdT, MAIT, NK CD56bright | 3 | 18 | 1.028 | 4.94e-16 | 0.641 | 7.14e-61 |
| RALGPS2 | Ral GEF with PH domain and SH3 binding motif 2 | B Naive, B Intermediate, B Memory | 3 | 18 | 1.056 | 3.20e-10 | 1.466 | 0.00e+00 |
| BLK | BLK proto-oncogene, Src family tyrosine kinase | B Naive, B Intermediate, B Memory | 3 | 18 | 3.651 | 1.27e-112 | 1.132 | 0.00e+00 |
| PLEKHG1 | pleckstrin homology and RhoGEF domain containing G1 | B Naive, B Intermediate | 2 | 18 | 2.643 | 9.62e-125 | 0.988 | 0.00e+00 |
| RUNX3 | RUNX family transcription factor 3 | NK CD56bright | 1 | 17 | 0.751 | 1.50e-04 | 0.781 | 2.58e-50 |
| EZR | ezrin | B Naive, B Intermediate, B Memory | 3 | 16 | 0.510 | 8.11e-03 | 0.784 | 2.48e-156 |
| EVI5 | ecotropic viral integration site 5 | CD14 Mono, CD16 Mono | 2 | 16 | 1.193 | 5.39e-113 | 0.702 | 2.50e-234 |
| FOXP1 | forkhead box P1 | Treg, B Naive, CD8 Naive | 3 | 15 | 0.574 | 2.37e-06 | 0.564 | 2.10e-84 |
| SULF2 | sulfatase 2 | CD14 Mono, pDC | 2 | 14 | 1.344 | 2.61e-127 | 0.781 | 8.66e-244 |
| ZFP36L2 | ZFP36 ring finger protein like 2 | MAIT, CD8 TCM/TEM | 2 | 14 | 0.378 | 1.67e-03 | 0.508 | 3.41e-30 |
| BCL11A | BAF chromatin remodeling complex subunit BCL11A | B Naive, B Intermediate, pDC | 3 | 13 | 1.973 | 2.53e-41 | 0.858 | 0.00e+00 |
| ABLIM1 | actin binding LIM protein 1 | CD8 Naive | 1 | 13 | 0.512 | 2.86e-17 | 0.508 | 1.45e-218 |
| SWAP70 | switching B cell complex subunit SWAP70 | B Naive, B Intermediate, B Memory | 3 | 12 | 1.213 | 6.14e-24 | 0.601 | 0.00e+00 |
| PTPRE | protein tyrosine phosphatase receptor type E | CD14 Mono, pDC | 2 | 12 | 1.048 | 6.79e-151 | 0.800 | 0.00e+00 |
| LARGE1 | LARGE xylosyl- and glucuronyltransferase 1 | B Naive, B Memory | 2 | 11 | 1.942 | 1.02e-62 | 0.987 | 0.00e+00 |
| SNX9 | sorting nexin 9 | B Naive | 1 | 11 | 0.711 | 7.83e-11 | 0.845 | 1.18e-151 |
| PTPN6 | protein tyrosine phosphatase non-receptor type 6 | CD16 Mono | 1 | 11 | 0.535 | 4.96e-01 | 0.538 | 2.47e-164 |
| CD69 | CD69 molecule | B Naive | 1 | 11 | 0.410 | 1.62e-01 | 0.987 | 5.38e-117 |
| RABAC1 | Rab acceptor 1 | Plasmablast | 1 | 11 | 0.766 | 1.00e+00 | 0.659 | 3.25e-37 |
| RNF149 | ring finger protein 149 | CD16 Mono, pDC | 2 | 10 | 0.492 | 3.80e-01 | 0.660 | 5.50e-58 |
| VOPP1 | VOPP1 WW domain binding protein | B Intermediate, Plasmablast | 2 | 10 | 2.162 | 1.54e-31 | 0.601 | 6.37e-88 |
| P2RX1 | purinergic receptor P2X 1 | pDC | 1 | 10 | 1.702 | 3.53e-22 | 0.677 | 4.66e-174 |
| SLC15A4 | solute carrier family 15 member 4 | pDC | 1 | 10 | 0.406 | 1.00e+00 | 0.963 | 6.13e-269 |
| FOS | Fos proto-oncogene, AP-1 transcription factor subunit | MAIT | 1 | 10 | 0.280 | 1.00e+00 | 0.632 | 8.13e-45 |
| DUSP2 | dual specificity phosphatase 2 | gdT, MAIT, NK CD56bright | 3 | 9 | 0.385 | 9.77e-01 | 0.759 | 2.91e-87 |
| MEF2C | myocyte enhancer factor 2C | B Naive, B Intermediate, B Memory | 3 | 9 | 2.588 | 9.32e-66 | 1.146 | 0.00e+00 |
| CD37 | CD37 molecule | B Naive, B Intermediate, B Memory | 3 | 9 | 3.282 | 6.96e-119 | 0.964 | 1.15e-190 |
| BANK1 | B cell scaffold protein with ankyrin repeats 1 | B Naive, B Intermediate, B Memory | 3 | 9 | 2.898 | 6.76e-87 | 2.599 | 0.00e+00 |
| MS4A1 | membrane spanning 4-domains A1 | B Naive, B Intermediate, B Memory | 3 | 9 | 4.101 | 5.08e-149 | 1.438 | 0.00e+00 |
| SEL1L3 | SEL1L family member 3 | B Intermediate, pDC, Plasmablast | 3 | 9 | 1.369 | 1.66e-09 | 0.982 | 3.57e-198 |
| PRKCB | protein kinase C beta | B Naive, B Intermediate, B Memory | 3 | 9 | 1.439 | 1.20e-24 | 0.815 | 2.08e-104 |
| VMP1 | vacuole membrane protein 1 | CD14 Mono, CD16 Mono | 2 | 9 | 0.519 | 1.13e-24 | 0.876 | 8.65e-185 |
| MAP3K8 | mitogen-activated protein kinase kinase kinase 8 | NK CD56bright | 1 | 9 | 0.543 | 6.30e-05 | 0.736 | 5.45e-62 |
| CD2AP | CD2 associated protein | pDC | 1 | 9 | 0.581 | 3.59e-01 | 1.435 | 3.91e-246 |
| ARHGAP26 | Rho GTPase activating protein 26 | CD14 Mono | 1 | 9 | 0.975 | 3.36e-128 | 1.374 | 1.92e-288 |
| C12orf75 | chromosome 12 open reading frame 75 | pDC | 1 | 9 | 0.521 | 1.00e+00 | 0.758 | 1.24e-147 |
| DENND5B | DENN domain containing 5B | B Intermediate, Plasmablast | 2 | 8 | 2.686 | 4.66e-46 | 0.905 | 0.00e+00 |
| CDK14 | cyclin dependent kinase 14 | B Naive, B Intermediate | 2 | 8 | 2.385 | 4.41e-91 | 1.122 | 0.00e+00 |
| SPOCK2 | SPARC (osteonectin), cwcv and kazal like domains proteoglycan 2 | MAIT | 1 | 8 | 0.768 | 9.05e-09 | 0.550 | 1.08e-65 |
| IKZF2 | IKAROS family zinc finger 2 | Treg | 1 | 8 | 0.796 | 7.99e-08 | 0.736 | 1.60e-274 |
| S100A11 | S100 calcium binding protein A11 | CD16 Mono | 1 | 8 | 0.653 | 6.21e-06 | 0.894 | 2.74e-181 |
| CUX1 | cut like homeobox 1 | CD14 Mono | 1 | 8 | 0.699 | 4.54e-69 | 0.618 | 2.66e-130 |
| SLC1A4 | solute carrier family 1 member 4 | pDC | 1 | 8 | 0.823 | 4.13e-04 | 0.504 | 5.75e-162 |
| PILRA | paired immunoglobin like type 2 receptor alpha | CD16 Mono | 1 | 8 | 1.819 | 2.71e-39 | 0.718 | 0.00e+00 |
| SYTL3 | synaptotagmin like 3 | NK CD56bright | 1 | 8 | 0.372 | 1.00e+00 | 0.520 | 6.30e-22 |
| SYNE2 | spectrin repeat containing nuclear envelope protein 2 | gdT | 1 | 8 | 0.352 | 1.00e+00 | 0.571 | 4.89e-39 |
| KLF4 | KLF transcription factor 4 | CD14 Mono | 1 | 8 | 2.928 | 0.00e+00 | 0.657 | 1.14e-231 |
| AREG | amphiregulin | NK CD56bright | 1 | 7 | 0.595 | 9.96e-06 | 0.728 | 6.89e-94 |
| MCTP2 | multiple C2 and transmembrane domain containing 2 | NK CD56bright | 1 | 7 | 0.840 | 8.67e-13 | 0.843 | 6.20e-74 |
| EML4 | EMAP like 4 | MAIT | 1 | 7 | 0.823 | 3.00e-14 | 0.579 | 1.34e-55 |
| CAMK4 | calcium/calmodulin dependent protein kinase IV | CD8 Naive | 1 | 7 | 0.591 | 1.98e-18 | 0.591 | 3.02e-267 |
| TBC1D9 | TBC1 domain family member 9 | B Intermediate, B Memory | 2 | 6 | 2.628 | 4.81e-48 | 0.685 | 0.00e+00 |
| GNAQ | G protein subunit alpha q | pDC, HSPC | 2 | 6 | 1.178 | 4.44e-04 | 0.782 | 4.65e-23 |
| KHDRBS2 | KH RNA binding domain containing, signal transduction associated 2 | B Naive, B Intermediate | 2 | 6 | 3.018 | 4.16e-103 | 0.916 | 0.00e+00 |
| DOCK8 | dedicator of cytokinesis 8 | CD14 Mono, CD16 Mono | 2 | 6 | 2.669 | 0.00e+00 | 0.583 | 2.29e-65 |
| RFTN1 | raftlin, lipid raft linker 1 | pDC | 1 | 6 | 0.791 | 7.28e-02 | 0.586 | 4.70e-37 |
| CD79A | CD79a molecule | B Naive | 1 | 6 | 0.477 | 7.25e-04 | 1.518 | 0.00e+00 |
| NKG7 | natural killer cell granule protein 7 | NK CD56bright | 1 | 6 | 1.543 | 7.09e-41 | 0.621 | 5.03e-49 |
| STRBP | spermatid perinuclear RNA binding protein | B Naive | 1 | 6 | 0.284 | 6.85e-02 | 0.870 | 0.00e+00 |
| CHST15 | carbohydrate sulfotransferase 15 | CD16 Mono | 1 | 6 | 2.586 | 4.72e-59 | 0.539 | 0.00e+00 |
| MANBA | mannosidase beta | CD14 Mono | 1 | 6 | 0.698 | 4.53e-76 | 0.525 | 3.04e-108 |
| TNFRSF13C | TNF receptor superfamily member 13C | B Intermediate | 1 | 6 | 1.590 | 2.44e-41 | 0.542 | 0.00e+00 |
| ARHGEF40 | Rho guanine nucleotide exchange factor 40 | CD14 Mono | 1 | 6 | 0.569 | 1.68e-48 | 0.504 | 8.11e-282 |
| IER2 | immediate early response 2 | NK CD56bright | 1 | 6 | 0.834 | 1.45e-07 | 0.729 | 2.31e-37 |
| SAMHD1 | SAM and HD domain containing deoxynucleoside triphosphate triphosphohydrolase 1 | CD14 Mono | 1 | 6 | 0.338 | 1.29e-13 | 0.518 | 8.40e-67 |
| ARAP2 | ArfGAP with RhoGAP domain, ankyrin repeat and PH domain 2 | NK CD56bright | 1 | 6 | 0.595 | 1.23e-04 | 0.585 | 6.26e-32 |
| TAGLN2 | transgelin 2 | HSPC | 1 | 6 | 0.935 | 1.00e+00 | 0.658 | 1.84e-04 |
| APP | amyloid beta precursor protein | pDC | 1 | 6 | 0.699 | 1.00e+00 | 2.000 | 0.00e+00 |
| UBE2J1 | ubiquitin conjugating enzyme E2 J1 | Plasmablast | 1 | 6 | 0.682 | 1.00e+00 | 1.061 | 9.28e-97 |
| SLC41A2 | solute carrier family 41 member 2 | pDC | 1 | 6 | 0.642 | 1.00e+00 | 0.656 | 0.00e+00 |
| PMEPA1 | prostate transmembrane protein, androgen induced 1 | pDC | 1 | 6 | 0.573 | 1.00e+00 | 0.555 | 1.85e-231 |
| IRF7 | interferon regulatory factor 7 | pDC | 1 | 6 | 0.405 | 1.00e+00 | 0.557 | 6.82e-176 |
| SRGN | serglycin | NK CD56bright | 1 | 6 | 0.316 | 1.00e+00 | 0.577 | 2.49e-20 |
| PARP8 | poly(ADP-ribose) polymerase family member 8 | NK CD56bright | 1 | 6 | 0.266 | 1.00e+00 | 0.544 | 2.77e-15 |
| TNFAIP3 | TNF alpha induced protein 3 | MAIT, CD8 TCM/TEM | 2 | 5 | 0.323 | 7.58e-13 | 0.572 | 5.97e-39 |
| SOX4 | SRY-box transcription factor 4 | pDC, HSPC | 2 | 5 | 2.041 | 2.11e-03 | 1.390 | 1.15e-134 |
| ST6GAL1 | ST6 beta-galactoside alpha-2,6-sialyltransferase 1 | B Naive, B Intermediate | 2 | 5 | 0.490 | 1.96e-01 | 0.591 | 1.68e-91 |
| PRKCE | protein kinase C epsilon | B Naive, B Intermediate | 2 | 5 | 3.313 | 1.69e-116 | 1.134 | 0.00e+00 |
| HLA-DQB1 | major histocompatibility complex, class II, DQ beta 1 | B Naive, B Intermediate | 2 | 5 | 1.948 | 1.46e-42 | 1.139 | 0.00e+00 |
| LDLRAD4 | low density lipoprotein receptor class A domain containing 4 | Treg, pDC | 2 | 5 | 1.727 | 1.38e-18 | 0.921 | 7.93e-64 |
| RNF130 | ring finger protein 130 | CD14 Mono | 1 | 5 | 0.304 | 6.36e-12 | 0.843 | 1.32e-284 |
| MZB1 | marginal zone B and B1 cell specific protein | Plasmablast | 1 | 5 | 1.638 | 5.95e-06 | 2.713 | 0.00e+00 |
| EMILIN2 | elastin microfibril interfacer 2 | CD16 Mono | 1 | 5 | 1.380 | 5.86e-17 | 0.585 | 3.16e-183 |
| MARCKS | myristoylated alanine rich protein kinase C substrate | B Memory | 1 | 5 | 2.241 | 3.52e-53 | 0.507 | 4.67e-104 |
| IFI30 | IFI30 lysosomal thiol reductase | CD16 Mono | 1 | 5 | 1.655 | 2.76e-44 | 1.361 | 0.00e+00 |
| NFKB1 | nuclear factor kappa B subunit 1 | NK CD56bright | 1 | 5 | 0.494 | 2.52e-04 | 0.671 | 3.77e-26 |
| SH2B3 | SH2B adaptor protein 3 | pDC | 1 | 5 | 0.820 | 1.16e-01 | 1.107 | 3.01e-203 |
| MAFB | MAF bZIP transcription factor B | CD16 Mono | 1 | 5 | 2.351 | 1.02e-67 | 0.577 | 0.00e+00 |
| DENND1B | DENN domain containing 1B | Plasmablast | 1 | 5 | 0.725 | 1.00e+00 | 0.790 | 7.46e-29 |
| MYO1D | myosin ID | Plasmablast | 1 | 5 | 0.639 | 1.00e+00 | 1.573 | 4.74e-271 |
| RORA | RAR related orphan receptor A | gdT | 1 | 5 | 0.378 | 1.00e+00 | 0.764 | 6.23e-52 |
| TBC1D5 | TBC1 domain family member 5 | B Intermediate | 1 | 5 | 0.290 | 1.00e+00 | 0.524 | 2.43e-50 |
| JAZF1 | JAZF zinc finger 1 | B Naive | 1 | 5 | 0.263 | 1.00e+00 | 0.551 | 1.73e-95 |
| COBLL1 | cordon-bleu WH2 repeat protein like 1 | B Naive, B Intermediate | 2 | 4 | 3.020 | 9.14e-91 | 0.887 | 0.00e+00 |
| NCOA3 | nuclear receptor coactivator 3 | B Intermediate, Plasmablast | 2 | 4 | 1.454 | 8.63e-12 | 0.983 | 2.54e-88 |
| HLA-DPB1 | major histocompatibility complex, class II, DP beta 1 | B Naive, B Intermediate | 2 | 4 | 0.943 | 8.50e-15 | 1.116 | 0.00e+00 |
| HLA-DPA1 | major histocompatibility complex, class II, DP alpha 1 | B Intermediate, B Memory | 2 | 4 | 1.615 | 4.62e-21 | 1.220 | 0.00e+00 |
| DMXL2 | Dmx like 2 | CD14 Mono, CD16 Mono | 2 | 4 | 1.669 | 4.32e-139 | 0.880 | 0.00e+00 |
| ADAM28 | ADAM metallopeptidase domain 28 | B Naive, B Memory | 2 | 4 | 4.262 | 3.39e-119 | 0.955 | 0.00e+00 |
| HLA-DRA | major histocompatibility complex, class II, DR alpha | B Naive, B Memory | 2 | 4 | 1.706 | 2.23e-31 | 1.979 | 0.00e+00 |
| FCRL1 | Fc receptor like 1 | B Naive, B Memory | 2 | 4 | 4.452 | 2.18e-122 | 0.953 | 0.00e+00 |
| CD79B | CD79b molecule | B Intermediate, B Memory | 2 | 4 | 1.953 | 1.63e-29 | 0.775 | 0.00e+00 |
| CCSER1 | coiled-coil serine rich protein 1 | B Naive, B Intermediate | 2 | 4 | 2.780 | 1.12e-89 | 1.020 | 0.00e+00 |
| ARHGAP24 | Rho GTPase activating protein 24 | B Intermediate, pDC | 2 | 4 | 3.031 | 1.05e-63 | 1.319 | 0.00e+00 |
| PLXDC2 | plexin domain containing 2 | CD14 Mono, CD16 Mono | 2 | 4 | 3.306 | 0.00e+00 | 1.099 | 0.00e+00 |
| SIPA1L1 | signal induced proliferation associated 1 like 1 | B Naive | 1 | 4 | 1.635 | 8.23e-54 | 0.827 | 4.05e-168 |
| ADAM17 | ADAM metallopeptidase domain 17 | CD14 Mono | 1 | 4 | 0.429 | 3.30e-34 | 0.529 | 6.38e-105 |
| RPS9 | ribosomal protein S9 | HSPC | 1 | 4 | 1.928 | 2.60e-01 | 0.641 | 4.49e-03 |
| RTKN2 | rhotekin 2 | Treg | 1 | 4 | 1.151 | 2.30e-20 | 0.507 | 0.00e+00 |
| POU2AF1 | POU class 2 homeobox associating factor 1 | Plasmablast | 1 | 4 | 2.055 | 1.71e-08 | 0.742 | 0.00e+00 |
| ANXA5 | annexin A5 | CD16 Mono | 1 | 4 | 0.634 | 1.47e-01 | 0.513 | 7.53e-127 |
| CCDC171 | coiled-coil domain containing 171 | HSPC | 1 | 4 | 0.996 | 1.00e+00 | 0.728 | 1.43e-85 |
| DERL3 | derlin 3 | Plasmablast | 1 | 4 | 0.568 | 1.00e+00 | 1.299 | 0.00e+00 |
| SP140 | SP140 nuclear body protein | B Intermediate | 1 | 4 | 0.414 | 1.00e+00 | 0.561 | 4.69e-131 |
| NCALD | neurocalcin delta | NK CD56bright | 1 | 3 | 1.732 | 8.49e-30 | 1.149 | 1.56e-115 |
| PTPRS | protein tyrosine phosphatase receptor type S | pDC | 1 | 3 | 2.532 | 7.28e-29 | 1.349 | 0.00e+00 |
| ANTXR2 | ANTXR cell adhesion molecule 2 | pDC | 1 | 3 | 0.923 | 6.68e-02 | 0.661 | 2.52e-58 |
| GLS | glutaminase | Plasmablast | 1 | 3 | 0.938 | 5.55e-01 | 0.505 | 7.10e-14 |
| CD68 | CD68 molecule | CD16 Mono | 1 | 3 | 1.250 | 5.09e-16 | 0.721 | 0.00e+00 |
| ISG20 | interferon stimulated exonuclease gene 20 | Plasmablast | 1 | 3 | 1.328 | 4.42e-02 | 0.927 | 1.28e-47 |
| STX7 | syntaxin 7 | B Naive | 1 | 3 | 0.764 | 4.16e-23 | 0.507 | 4.22e-173 |
| ADGRE2 | adhesion G protein-coupled receptor E2 | CD16 Mono | 1 | 3 | 2.345 | 3.92e-38 | 0.557 | 2.14e-240 |
| IKZF3 | IKAROS family zinc finger 3 | B Intermediate | 1 | 3 | 1.436 | 3.87e-27 | 0.562 | 5.06e-115 |
| NDFIP1 | Nedd4 family interacting protein 1 | CD8 Naive | 1 | 3 | 0.879 | 3.56e-33 | 0.684 | 0.00e+00 |
| UBE2E2 | ubiquitin conjugating enzyme E2 E2 | B Naive | 1 | 3 | 1.349 | 3.33e-35 | 0.772 | 1.88e-199 |
| ST6GALNAC3 | ST6 N-acetylgalactosaminide alpha-2,6-sialyltransferase 3 | B Naive | 1 | 3 | 0.794 | 2.73e-11 | 0.618 | 0.00e+00 |
| SP100 | SP100 nuclear antigen | B Naive | 1 | 3 | 3.156 | 2.49e-131 | 0.511 | 1.39e-72 |
| COL19A1 | collagen type XIX alpha 1 chain | B Naive | 1 | 3 | 1.998 | 2.32e-74 | 1.321 | 0.00e+00 |
| STX11 | syntaxin 11 | CD14 Mono | 1 | 3 | 1.334 | 2.16e-235 | 0.588 | 1.76e-195 |
| TMTC2 | transmembrane O-mannosyltransferase targeting cadherins 2 | CD14 Mono | 1 | 3 | 0.501 | 2.12e-28 | 0.544 | 6.44e-126 |
| OAZ1 | ornithine decarboxylase antizyme 1 | CD16 Mono | 1 | 3 | 0.970 | 1.73e-11 | 0.695 | 1.67e-83 |
| RILPL2 | Rab interacting lysosomal protein like 2 | CD14 Mono | 1 | 3 | 0.591 | 1.58e-65 | 0.789 | 1.41e-202 |
| ACTB | actin beta | CD16 Mono | 1 | 3 | 0.934 | 1.49e-09 | 1.419 | 5.59e-185 |
| PIP5K1B | phosphatidylinositol-4-phosphate 5-kinase type 1 beta | B Intermediate | 1 | 3 | 2.999 | 1.46e-101 | 0.571 | 0.00e+00 |
| SSPN | sarcospan | B Memory | 1 | 3 | 4.484 | 1.40e-84 | 0.778 | 0.00e+00 |
| PHTF1 | putative homeodomain transcription factor 1 | HSPC | 1 | 3 | 1.044 | 1.00e+00 | 1.040 | 9.77e-29 |
| ANKRD11 | ankyrin repeat domain containing 11 | pDC | 1 | 3 | 0.650 | 1.00e+00 | 0.642 | 3.04e-34 |
| LMAN1 | lectin, mannose binding 1 | Plasmablast | 1 | 3 | 0.644 | 1.00e+00 | 0.969 | 9.67e-117 |
| ANKRD28 | ankyrin repeat domain 28 | Plasmablast | 1 | 3 | 0.622 | 1.00e+00 | 0.652 | 2.86e-20 |
| PDIA3 | protein disulfide isomerase family A member 3 | Plasmablast | 1 | 3 | 0.597 | 1.00e+00 | 0.644 | 4.30e-22 |
| LYPLAL1 | lysophospholipase like 1 | B Memory | 1 | 3 | 0.562 | 1.00e+00 | 0.647 | 1.29e-104 |
| RGS2 | regulator of G protein signaling 2 | pDC | 1 | 3 | 0.507 | 1.00e+00 | 0.545 | 2.61e-37 |
| FUT8 | fucosyltransferase 8 | Plasmablast | 1 | 3 | 0.503 | 1.00e+00 | 1.261 | 3.66e-59 |
| ELF2 | E74 like ETS transcription factor 2 | pDC | 1 | 3 | 0.493 | 1.00e+00 | 0.517 | 1.45e-27 |
| DUSP1 | dual specificity phosphatase 1 | CD16 Mono | 1 | 3 | 0.342 | 1.00e+00 | 0.645 | 5.78e-55 |
| RBM47 | RNA binding motif protein 47 | CD14 Mono | 1 | 3 | 4.084 | 0.00e+00 | 1.068 | 0.00e+00 |
| RAB31 | RAB31, member RAS oncogene family | CD16 Mono | 1 | 2 | 1.991 | 9.65e-31 | 0.577 | 3.58e-170 |
| SEC61B | SEC61 translocon subunit beta | pDC | 1 | 2 | 4.142 | 8.26e-57 | 0.617 | 2.06e-62 |
| VASP | vasodilator stimulated phosphoprotein | CD16 Mono | 1 | 2 | 1.736 | 7.83e-25 | 0.538 | 2.51e-150 |
| CHD9 | chromodomain helicase DNA binding protein 9 | pDC | 1 | 2 | 1.586 | 7.20e-12 | 0.816 | 2.03e-92 |
| IL4R | interleukin 4 receptor | B Naive | 1 | 2 | 0.496 | 6.31e-02 | 0.705 | 3.29e-246 |
| CEBPB | CCAAT enhancer binding protein beta | CD16 Mono | 1 | 2 | 0.543 | 5.44e-02 | 0.516 | 4.27e-126 |
| ETV6 | ETS variant transcription factor 6 | pDC | 1 | 2 | 2.923 | 4.36e-38 | 0.885 | 1.05e-61 |
| GAB1 | GRB2 associated binding protein 1 | Plasmablast | 1 | 2 | 2.612 | 4.31e-16 | 1.153 | 1.01e-225 |
| HIVEP1 | HIVEP zinc finger 1 | pDC | 1 | 2 | 0.804 | 4.04e-04 | 0.754 | 1.62e-70 |
| KLRF1 | killer cell lectin like receptor F1 | NK CD56bright | 1 | 2 | 0.557 | 3.95e-01 | 0.796 | 6.73e-170 |
| FCER1G | Fc epsilon receptor Ig | NK CD56bright | 1 | 2 | 1.668 | 2.78e-30 | 0.592 | 3.62e-76 |
| CD63 | CD63 molecule | NK CD56bright | 1 | 2 | 0.620 | 2.77e-02 | 0.559 | 1.52e-44 |
| RGS1 | regulator of G protein signaling 1 | pDC | 1 | 2 | 0.958 | 2.68e-02 | 0.948 | 5.28e-155 |
| FYTTD1 | forty-two-three domain containing 1 | pDC | 1 | 2 | 0.599 | 2.32e-02 | 0.529 | 4.32e-81 |
| AKAP13 | A-kinase anchoring protein 13 | pDC | 1 | 2 | 1.314 | 2.29e-07 | 0.759 | 2.27e-38 |
| CASC15 | cancer susceptibility 15 | HSPC | 1 | 2 | 2.848 | 2.08e-01 | 1.139 | 0.00e+00 |
| MPEG1 | macrophage expressed 1 | pDC | 1 | 2 | 2.971 | 2.00e-40 | 0.772 | 3.18e-219 |
| PLXNB2 | plexin B2 | CD16 Mono | 1 | 2 | 2.409 | 1.80e-46 | 0.650 | 0.00e+00 |
| HS3ST3B1 | heparan sulfate-glucosamine 3-sulfotransferase 3B1 | pDC | 1 | 2 | 2.218 | 1.40e-26 | 0.648 | 1.53e-177 |
| NFKBIZ | NFKB inhibitor zeta | CD16 Mono | 1 | 2 | 0.582 | 1.15e-06 | 0.737 | 9.12e-104 |
| ADGRB3 | adhesion G protein-coupled receptor B3 | pDC | 1 | 2 | 3.361 | 1.06e-40 | 0.590 | 2.68e-219 |
| XBP1 | X-box binding protein 1 | Plasmablast | 1 | 2 | 0.659 | 1.00e+00 | 1.226 | 9.54e-97 |
| HM13 | histocompatibility minor 13 | Plasmablast | 1 | 2 | 0.615 | 1.00e+00 | 0.647 | 1.76e-46 |
| LRMP |  | B Memory | 1 | 2 | 0.450 | 1.00e+00 | 0.506 | 2.81e-42 |
| MARCH3 |  | B Naive | 1 | 2 | 0.265 | 1.00e+00 | 0.644 | 3.01e-264 |
| FTH1 | ferritin heavy chain 1 | CD16 Mono | 1 | 2 | 0.264 | 1.00e+00 | 1.390 | 5.56e-193 |
| MGAT5 | alpha-1,6-mannosylglycoprotein 6-beta-N-acetylglucosaminyltransferase | B Naive | 1 | 2 | 0.251 | 1.00e+00 | 0.948 | 8.81e-220 |
| IL1B | interleukin 1 beta | CD14 Mono | 1 | 2 | 4.961 | 0.00e+00 | 0.858 | 0.00e+00 |
| SLC11A1 | solute carrier family 11 member 1 | CD14 Mono | 1 | 2 | 3.708 | 0.00e+00 | 0.806 | 0.00e+00 |
| SPIDR | scaffold protein involved in DNA repair | CD14 Mono | 1 | 2 | 2.931 | 0.00e+00 | 0.713 | 3.63e-119 |
| FNDC3B | fibronectin type III domain containing 3B | CD14 Mono | 1 | 2 | 2.399 | 0.00e+00 | 0.690 | 3.08e-112 |
| H3F3A |  | CD16 Mono | 1 | 1 | 2.608 | 9.46e-44 | 0.574 | 3.97e-47 |
| CMTM6 | CKLF like MARVEL transmembrane domain containing 6 | CD16 Mono | 1 | 1 | 0.581 | 7.31e-02 | 0.642 | 1.15e-131 |
| TCF7L2 | transcription factor 7 like 2 | CD16 Mono | 1 | 1 | 4.208 | 7.07e-103 | 1.864 | 0.00e+00 |
| ZFAT | zinc finger and AT-hook domain containing | pDC | 1 | 1 | 5.524 | 6.88e-84 | 1.712 | 0.00e+00 |
| LRRC25 | leucine rich repeat containing 25 | CD16 Mono | 1 | 1 | 2.577 | 6.82e-53 | 0.763 | 0.00e+00 |
| IFITM3 | interferon induced transmembrane protein 3 | CD16 Mono | 1 | 1 | 1.677 | 6.66e-35 | 1.100 | 0.00e+00 |
| CDC42BPA | CDC42 binding protein kinase alpha | HSPC | 1 | 1 | 3.862 | 6.48e-10 | 0.571 | 1.90e-125 |
| COTL1 | coactosin like F-actin binding protein 1 | CD16 Mono | 1 | 1 | 1.633 | 6.35e-26 | 1.523 | 0.00e+00 |
| RERE | arginine-glutamic acid dipeptide repeats | B Naive | 1 | 1 | 0.357 | 5.41e-03 | 0.562 | 8.04e-85 |
| TPM3 | tropomyosin 3 | CD16 Mono | 1 | 1 | 1.126 | 5.10e-12 | 0.542 | 2.44e-70 |
| ARID5B | AT-rich interaction domain 5B | B Memory | 1 | 1 | 4.137 | 4.89e-66 | 0.611 | 1.64e-46 |
| MED12L | mediator complex subunit 12L | HSPC | 1 | 1 | 3.700 | 4.83e-05 | 1.420 | 8.40e-235 |
| TXNDC5 | thioredoxin domain containing 5 | Plasmablast | 1 | 1 | 1.631 | 4.45e-08 | 2.881 | 0.00e+00 |
| TET2 | tet methylcytosine dioxygenase 2 | CD14 Mono | 1 | 1 | 0.257 | 4.43e-06 | 0.754 | 1.52e-169 |
| LAPTM5 | lysosomal protein transmembrane 5 | B Naive | 1 | 1 | 1.876 | 4.40e-51 | 0.793 | 5.89e-161 |
| SEMA3C | semaphorin 3C | HSPC | 1 | 1 | 2.932 | 3.84e-01 | 0.549 | 1.14e-16 |
| TRAM1 | translocation associated membrane protein 1 | Plasmablast | 1 | 1 | 1.154 | 3.71e-01 | 0.748 | 9.08e-56 |
| WARS |  | CD16 Mono | 1 | 1 | 0.484 | 3.64e-02 | 1.157 | 0.00e+00 |
| SIPA1L3 | signal induced proliferation associated 1 like 3 | B Intermediate | 1 | 1 | 3.916 | 3.60e-115 | 0.790 | 1.32e-231 |
| HES4 | hes family bHLH transcription factor 4 | CD16 Mono | 1 | 1 | 1.420 | 3.33e-18 | 0.579 | 0.00e+00 |
| CD8B | CD8b molecule | CD8 Naive | 1 | 1 | 2.147 | 3.30e-165 | 0.668 | 0.00e+00 |
| SCAMP5 | secretory carrier membrane protein 5 | pDC | 1 | 1 | 1.045 | 3.28e-04 | 0.547 | 0.00e+00 |
| KIAA1211 |  | HSPC | 1 | 1 | 3.430 | 3.22e-03 | 0.806 | 0.00e+00 |
| PBX1 | PBX homeobox 1 | HSPC | 1 | 1 | 4.471 | 3.12e-06 | 0.798 | 6.95e-284 |
| SLCO3A1 | solute carrier organic anion transporter family member 3A1 | CD14 Mono | 1 | 1 | 0.288 | 2.97e-12 | 0.799 | 2.14e-141 |
| TBC1D8 | TBC1 domain family member 8 | pDC | 1 | 1 | 2.075 | 2.89e-18 | 0.595 | 2.12e-102 |
| HMOX1 | heme oxygenase 1 | CD16 Mono | 1 | 1 | 1.625 | 2.83e-24 | 0.594 | 0.00e+00 |
| CD22 | CD22 molecule | B Intermediate | 1 | 1 | 2.296 | 2.71e-51 | 0.603 | 0.00e+00 |
| FAM160A1 |  | pDC | 1 | 1 | 1.046 | 2.70e-03 | 1.917 | 0.00e+00 |
| RRBP1 | ribosome binding protein 1 | pDC | 1 | 1 | 4.640 | 2.64e-81 | 0.644 | 1.19e-109 |
| ITGA9 | integrin subunit alpha 9 | HSPC | 1 | 1 | 2.942 | 2.37e-01 | 0.528 | 0.00e+00 |
| RAPGEF1 | Rap guanine nucleotide exchange factor 1 | CD16 Mono | 1 | 1 | 2.657 | 2.33e-45 | 0.596 | 1.44e-50 |
| ALCAM | activated leukocyte cell adhesion molecule | pDC | 1 | 1 | 0.926 | 2.28e-02 | 0.808 | 9.59e-154 |
| EBF1 | EBF transcription factor 1 | B Intermediate | 1 | 1 | 5.545 | 2.09e-163 | 1.777 | 0.00e+00 |
| ZCCHC7 | zinc finger CCHC-type containing 7 | B Naive | 1 | 1 | 5.161 | 1.96e-233 | 0.954 | 3.52e-198 |
| SLC20A1 | solute carrier family 20 member 1 | pDC | 1 | 1 | 5.273 | 1.93e-87 | 0.639 | 3.00e-81 |
| NAMPT | nicotinamide phosphoribosyltransferase | CD16 Mono | 1 | 1 | 2.494 | 1.88e-43 | 1.206 | 8.40e-202 |
| ZNF521 | zinc finger protein 521 | HSPC | 1 | 1 | 2.523 | 1.86e-01 | 0.780 | 0.00e+00 |
| CEP128 | centrosomal protein 128 | Plasmablast | 1 | 1 | 1.468 | 1.81e-03 | 0.778 | 1.01e-64 |
| SCN9A | sodium voltage-gated channel alpha subunit 9 | pDC | 1 | 1 | 4.590 | 1.80e-57 | 0.796 | 0.00e+00 |
| SPI1 | Spi-1 proto-oncogene | CD16 Mono | 1 | 1 | 3.047 | 1.70e-62 | 0.891 | 0.00e+00 |
| SERPINF1 | serpin family F member 1 | pDC | 1 | 1 | 2.206 | 1.67e-27 | 0.738 | 0.00e+00 |
| CARD11 | caspase recruitment domain family member 11 | pDC | 1 | 1 | 3.036 | 1.64e-40 | 0.582 | 6.09e-39 |
| AHR | aryl hydrocarbon receptor | CD14 Mono | 1 | 1 | 1.039 | 1.53e-116 | 0.732 | 6.47e-166 |
| RHOH | ras homolog family member H | B Naive | 1 | 1 | 1.253 | 1.48e-24 | 0.591 | 3.76e-81 |
| ATP2C1 | ATPase secretory pathway Ca2+ transporting 1 | HSPC | 1 | 1 | 2.988 | 1.47e-01 | 0.832 | 4.03e-20 |
| MED13L | mediator complex subunit 13L | Plasmablast | 1 | 1 | 3.569 | 1.42e-23 | 0.544 | 8.41e-12 |
| NKAIN2 | sodium/potassium transporting ATPase interacting 2 | HSPC | 1 | 1 | 5.837 | 1.40e-09 | 1.790 | 0.00e+00 |
| MEIS1 | Meis homeobox 1 | HSPC | 1 | 1 | 4.007 | 1.29e-05 | 1.053 | 0.00e+00 |
| PDE4D | phosphodiesterase 4D | B Memory | 1 | 1 | 4.117 | 1.27e-67 | 2.131 | 3.98e-174 |
| GNAI2 | G protein subunit alpha i2 | CD16 Mono | 1 | 1 | 1.934 | 1.23e-30 | 0.629 | 1.52e-89 |
| PPM1H | protein phosphatase, Mg2+/Mn2+ dependent 1H | HSPC | 1 | 1 | 3.312 | 1.12e-02 | 0.513 | 2.57e-110 |
| HIPK3 | homeodomain interacting protein kinase 3 | CD14 Mono | 1 | 1 | 0.344 | 1.02e-13 | 0.512 | 1.17e-98 |
| MSI2 | musashi RNA binding protein 2 | HSPC | 1 | 1 | 2.500 | 1.00e+00 | 1.245 | 3.32e-16 |
| RPS23 | ribosomal protein S23 | HSPC | 1 | 1 | 0.860 | 1.00e+00 | 0.869 | 5.39e-06 |
| CCDC88A | coiled-coil domain containing 88A | pDC | 1 | 1 | 0.652 | 1.00e+00 | 1.275 | 5.37e-256 |
| SLC44A1 | solute carrier family 44 member 1 | Plasmablast | 1 | 1 | 0.620 | 1.00e+00 | 0.506 | 1.52e-44 |
| POLB | DNA polymerase beta | pDC | 1 | 1 | 0.577 | 1.00e+00 | 0.666 | 2.08e-119 |
| SAT1 | spermidine/spermine N1-acetyltransferase 1 | CD16 Mono | 1 | 1 | 0.509 | 1.00e+00 | 1.813 | 9.69e-303 |
| CORO1C | coronin 1C | pDC | 1 | 1 | 0.348 | 1.00e+00 | 0.912 | 0.00e+00 |
| MEF2A | myocyte enhancer factor 2A | B Intermediate | 1 | 1 | 0.348 | 1.00e+00 | 0.660 | 1.68e-85 |
| ZDHHC17 | zinc finger DHHC-type palmitoyltransferase 17 | pDC | 1 | 1 | 0.324 | 1.00e+00 | 0.819 | 1.15e-118 |
| PTP4A2 | protein tyrosine phosphatase 4A2 | CD16 Mono | 1 | 1 | 0.314 | 1.00e+00 | 0.508 | 1.65e-62 |
| SNX2 | sorting nexin 2 | B Intermediate | 1 | 1 | 0.306 | 1.00e+00 | 0.585 | 2.52e-123 |
| GAS7 | growth arrest specific 7 | CD14 Mono | 1 | 1 | 3.731 | 0.00e+00 | 0.944 | 5.71e-301 |

**ATAC peaks:** The number of ATAC-seq peaks associated with each gene. These peaks represent regions of open chromatin near the gene, indicating areas of potential regulatory activity. **Avg log2FC (scRNA-seq):** The average log2 fold change in gene expression as determined by single-cell RNA sequencing (scRNAseq). This value represents the ratio of gene expression levels in Alopecia Areata (AA) samples compared to controls. A positive value indicates higher expression in AA, while a negative value indicates lower expression. ****log10 p val adj (scRNA-seq):**** The log10-transformed adjusted p-value for differential gene expression as determined by scRNAseq. This value indicates the statistical significance of the observed fold change after correcting for multiple comparisons. Lower values represent more statistically significant results. ****Avg log2FC (scATAC-seq):**** The average log2 fold change in chromatin accessibility as determined by single-cell ATAC sequencing (scATACseq). This value represents the ratio of chromatin accessibility (open regions) in AA samples compared to controls near each gene. A positive value indicates more open chromatin in AA, while a negative value indicates less. ****log10 p val adj (scATAC-seq)**:** The log10-transformed adjusted p-value for differential chromatin accessibility as determined by scATACseq. This value indicates the statistical significance of the observed fold change in chromatin accessibility after correcting for multiple comparisons. Lower values represent more statistically significant results.

**Supplementary Figures**

**Fig S1.**


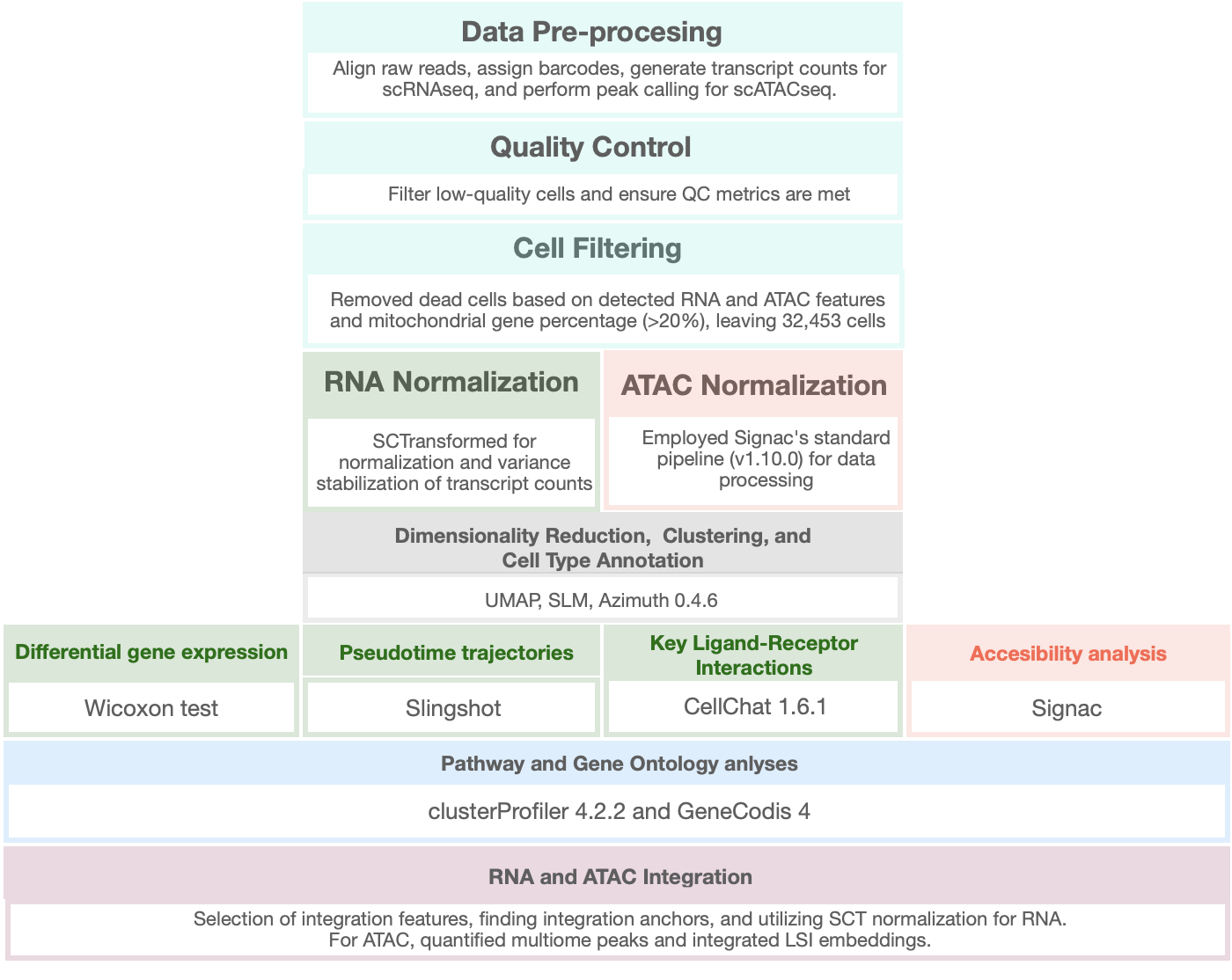


**Figure S1. Workflow diagram for the integrated scRNAseq/ATAC study in PBMCs.** The pipeline starts with sample collection and preparation, progressing through sequencing, data preprocessing, quality control, normalization, and dimensionality reduction. Following cell clustering, three parallel analysis pathways are represented: differential expression/accessibility analysis, pseudotime trajectories, and key ligand-receptor interactions. These analytical branches eventually converge for integrated functional enrichment, pathway analyses, and data visualization. The sequential steps are depicted with arrows, while parallel processes are branched and color-coded for clarity.

**Fig S2.**


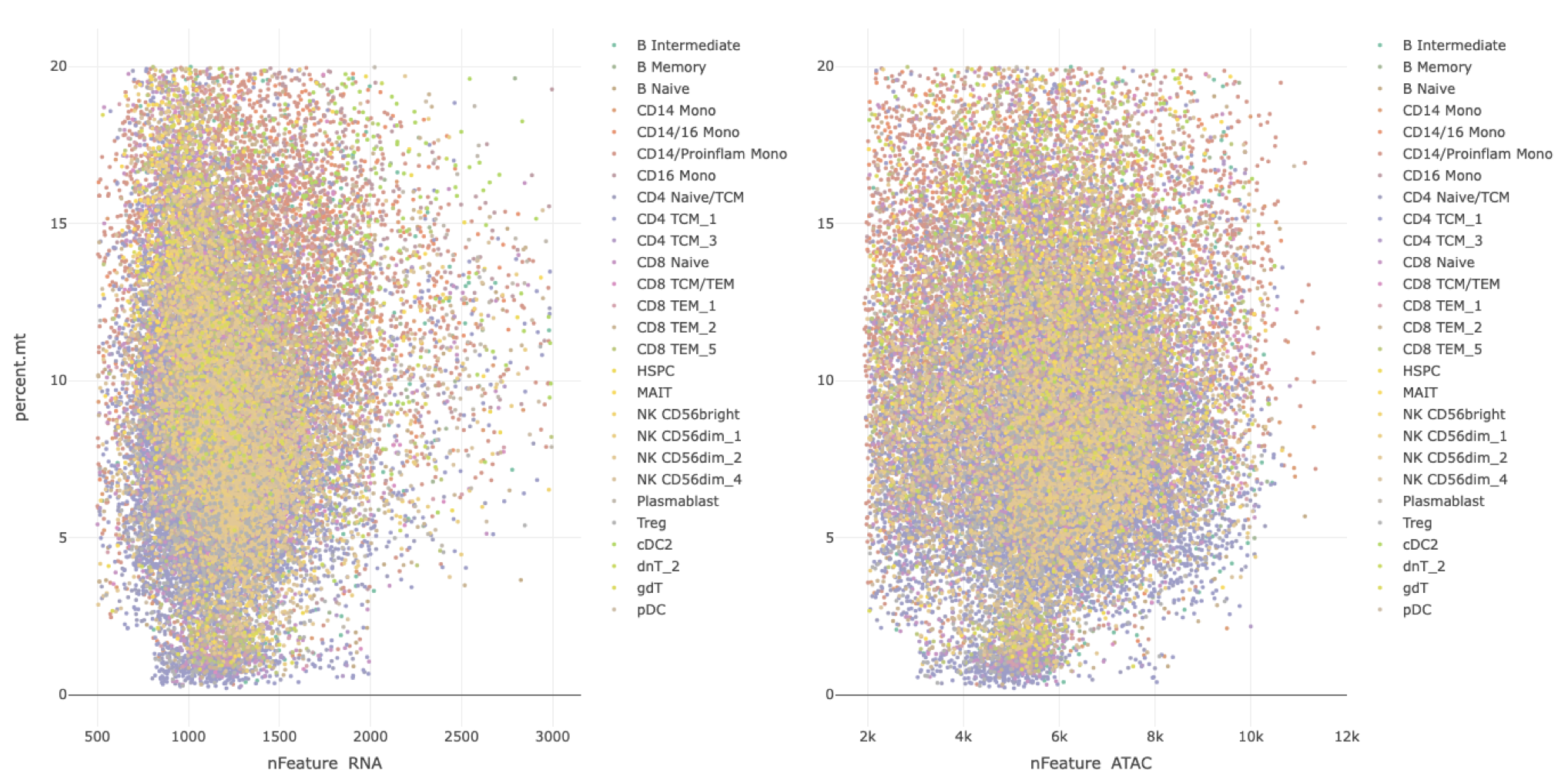


**Figure S2. Comparative Analysis of Gene Expression, Mitochondrial Genes, and Chromatin Accessibility Across Cell Subtypes.**

The left panel (A) displays the relationship between the number of detected genes per cell (nFeature_RNA) and mitochondrial gene percentage (percent.mt). The right panel (B) illustrates cell distribution based on unique accessible chromatin regions (nFeature_ATAC). Each dot represents an individual cell, color-coded by its subtype.

**Fig S3.**


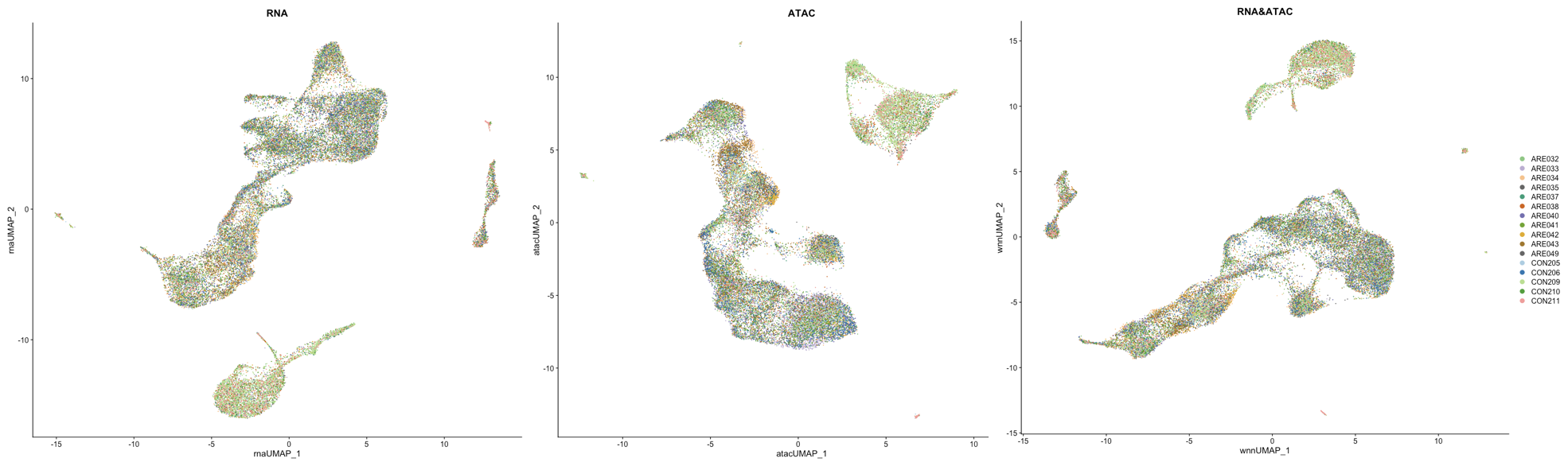


**Figure S3. Quality control of cell subtype contribution per subject in RNA, ATAC and RNA&ATAC analyses.**

**Fig S4.**


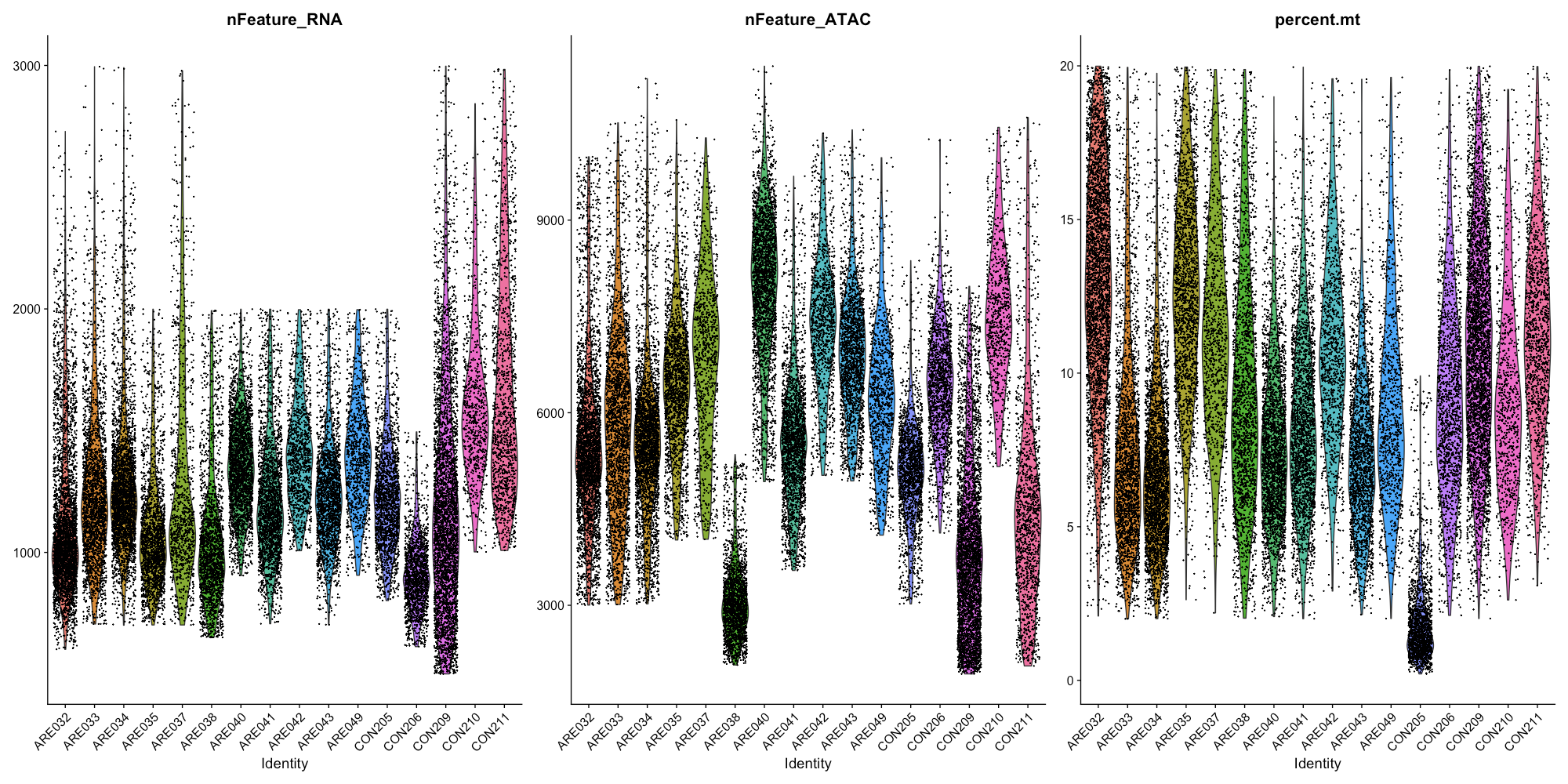


**Figure S4. Quality Control Metrics for scRNAseq and ATACseq of PBMCs from Alopecia Areata Patients and Healthy Controls.**

The provided quality control panel displays violin plots for three key metrics often utilized in single-cell RNA sequencing (scRNAseq) and ATAC sequencing (ATACseq) data processing. The plots contrast data from various patients with alopecia areata (denoted by “ARE") and healthy controls (denoted by “CON"). a) nFeature_RNA. Represents the number of unique RNA features (usually genes) detected in each cell. A higher count might indicate a cell with higher overall transcriptional activity or better sequencing depth. Conversely, a very low count might suggest a low-quality cell or one that was not well captured. The various colors represent different patient samples, and the width of the violin indicates the density of cells with a given number of features. b) nFeature_ATAC: Represents the number of unique chromatin accessibility regions detected in each cell. Like the RNA feature plot, a higher number might signify better accessibility or sequencing depth. The width of the violins suggests that there's variability in the number of accessible regions detected across the samples. c) percent.mt: Represents the percentage of mitochondrial genes detected in the RNA data of each cell. High mitochondrial gene expression can be a sign of cell stress or could indicate low-quality cells where the cytoplasmic RNA has degraded, leaving a disproportionate amount of mitochondrial RNA.Ideally, one might want to see lower percentages, and cells with very high mitochondrial percentages might be removed in quality control steps.

**Fig S5.**


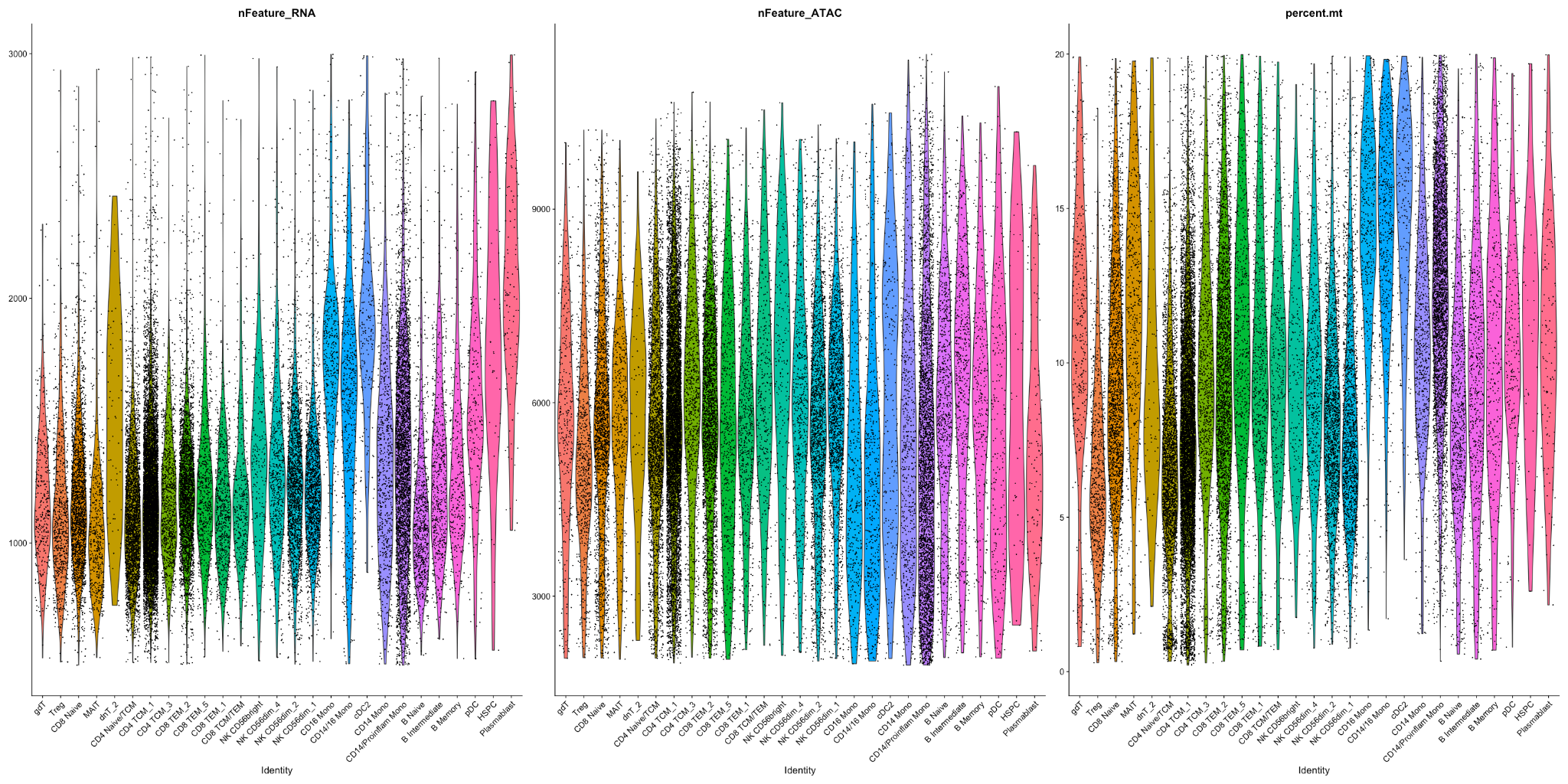


**Figure S5. Quality Control Metrics for scRNAseq and ATACseq of PBMCs across Various Cell Identities.**

The provided quality control panel displays violin plots for three key metrics often utilized in single-cell RNA sequencing (scRNAseq) and ATAC sequencing (ATACseq) data processing. The plots contrast data from various patients with alopecia areata (denoted by “ARE") and healthy controls (denoted by “CON"). a) nFeature_RNA. Represents the number of unique RNA features (usually genes) detected in each cell. A higher count might indicate a cell with higher overall transcriptional activity or better sequencing depth. Conversely, a very low count might suggest a low-quality cell or one that was not well captured. The various colors represent different patient samples, and the width of the violin indicates the density of cells with a given number of features. b) nFeature_ATAC: Represents the number of unique chromatin accessibility regions detected in each cell. Like the RNA feature plot, a higher number might signify better accessibility or sequencing depth. The width of the violins suggests that there's variability in the number of accessible regions detected across the samples. c) percent.mt: Represents the percentage of mitochondrial genes detected in the RNA data of each cell. High mitochondrial gene expression can be a sign of cell stress or could indicate low-quality cells where the cytoplasmic RNA has degraded, leaving a disproportionate amount of mitochondrial RNA.Ideally, one might want to see lower percentages, and cells with very high mitochondrial percentages might be removed in quality control steps.

**Fig S6.**


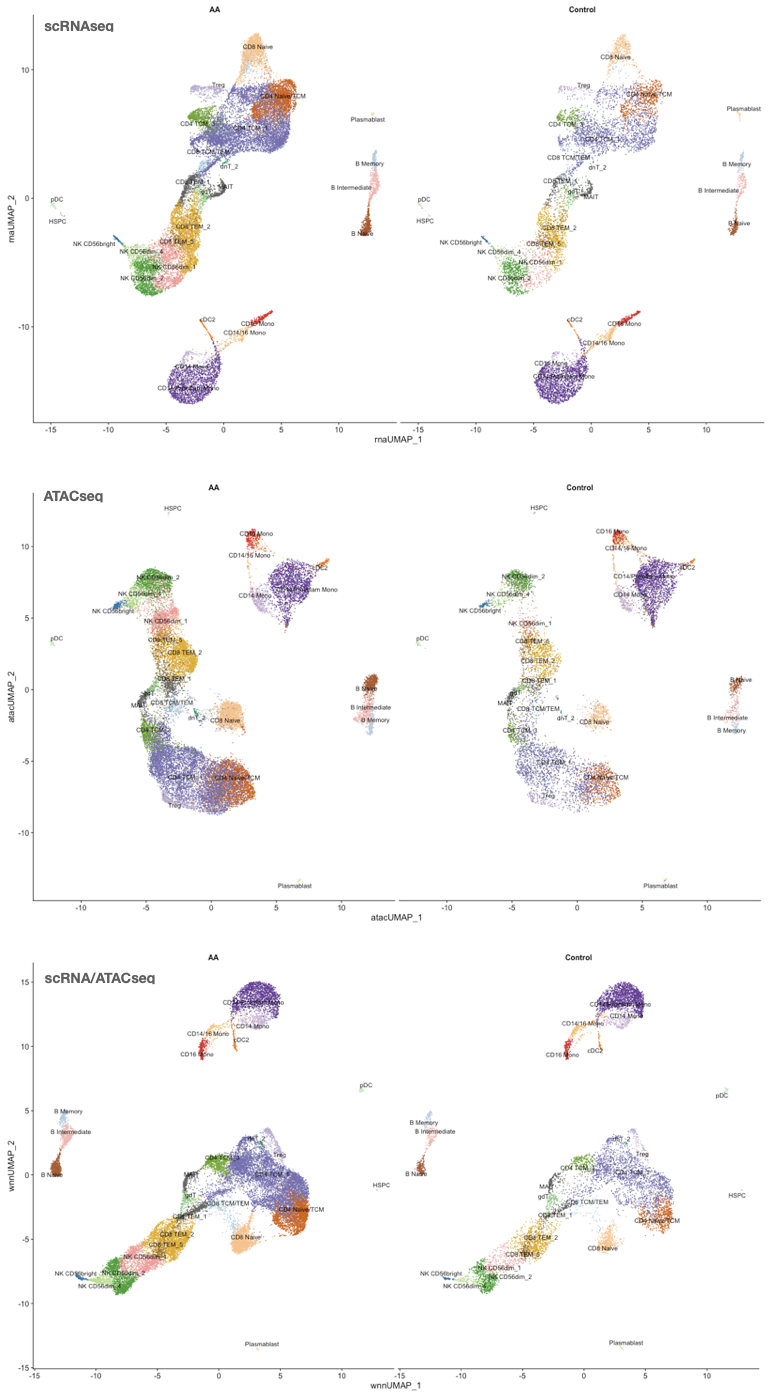


**Figure S6. Comparative UMAP plots of cellular clusters for "AA" vs. "Control" conditions across three datasets.** Rows represent different datasets: RNA sequencing (top), ATAC sequencing (middle), and integrated RNA & ATAC (bottom). Each dot signifies an individual cell, colored according to identified cellular clusters. Distinct cell types/subtypes, inferred from their respective profiles, are labeled. Both conditions display a diverse set of immune cells, although differences in cluster distribution and density can be observed.

**Fig S7.**


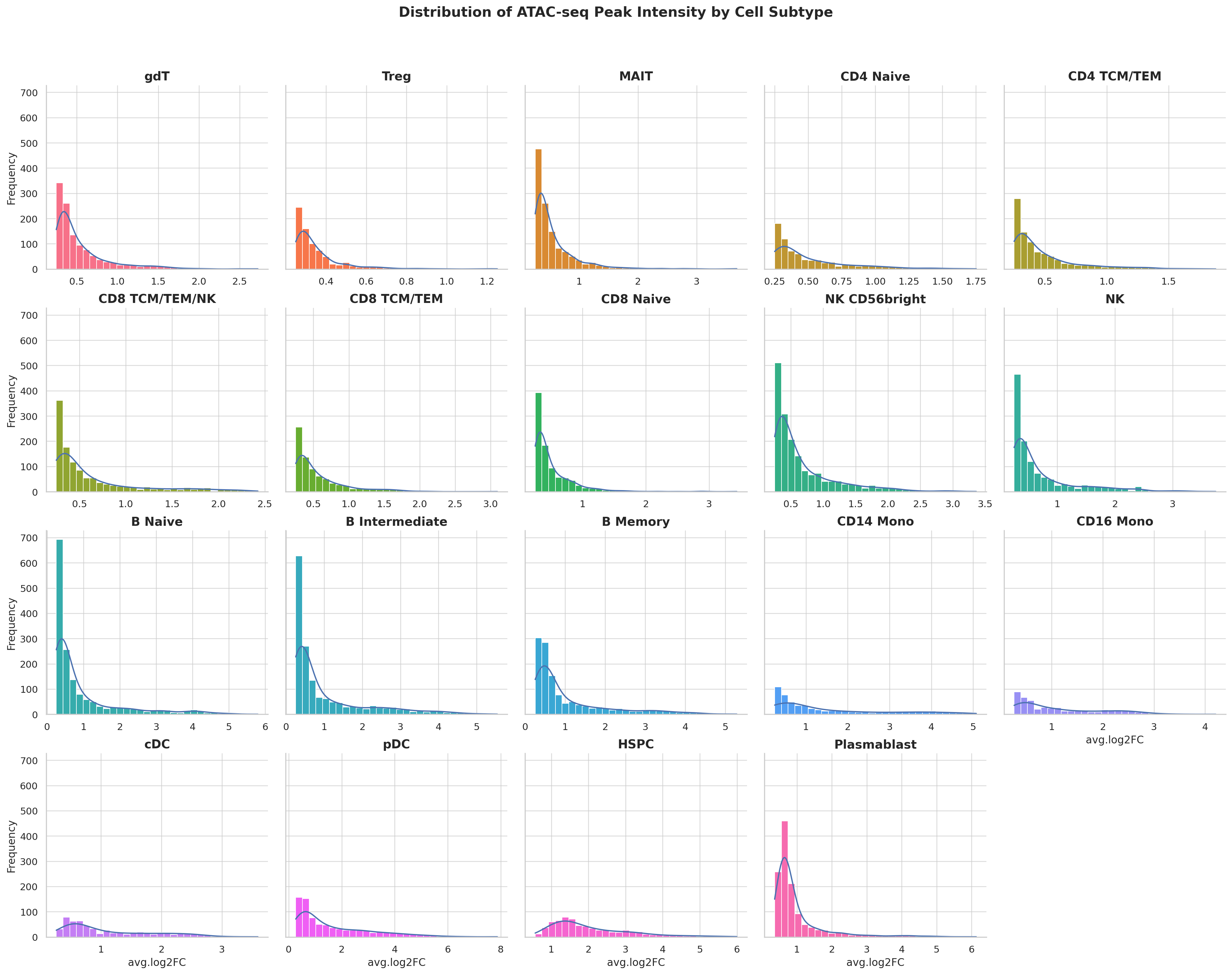


**Figure 6. Distribution of ATAC-seq Peak Intensity Across Immune Cell Subtypes.** This figure shows the distribution of ATAC-seq peak intensities, represented as average log2 fold change (avg.log2FC), across various immune cell subtypes. Each panel corresponds to a specific cell subtype, including gd T, Treg, MAIT, *CD4*^+^ naïve T cells, *CD4*^+^ TCM/TEM, *CD8*^+^ TCM/TEM/NK, *CD8*^+^ TCM/TEM, *CD8*^+^ Naive, NK *CD56^bright^*, NK, B naive, B Intermediate, B Memory, CD14^+^ Monocytes, *CD16*^+^ Monocytes, cDC,s pDCs, HSPC, and Plasmablasts. The histograms display the frequency distribution of ATAC-seq peaks within each cell type, providing insight into the chromatin accessibility landscape associated with these subtypes.

**Figure S8.**


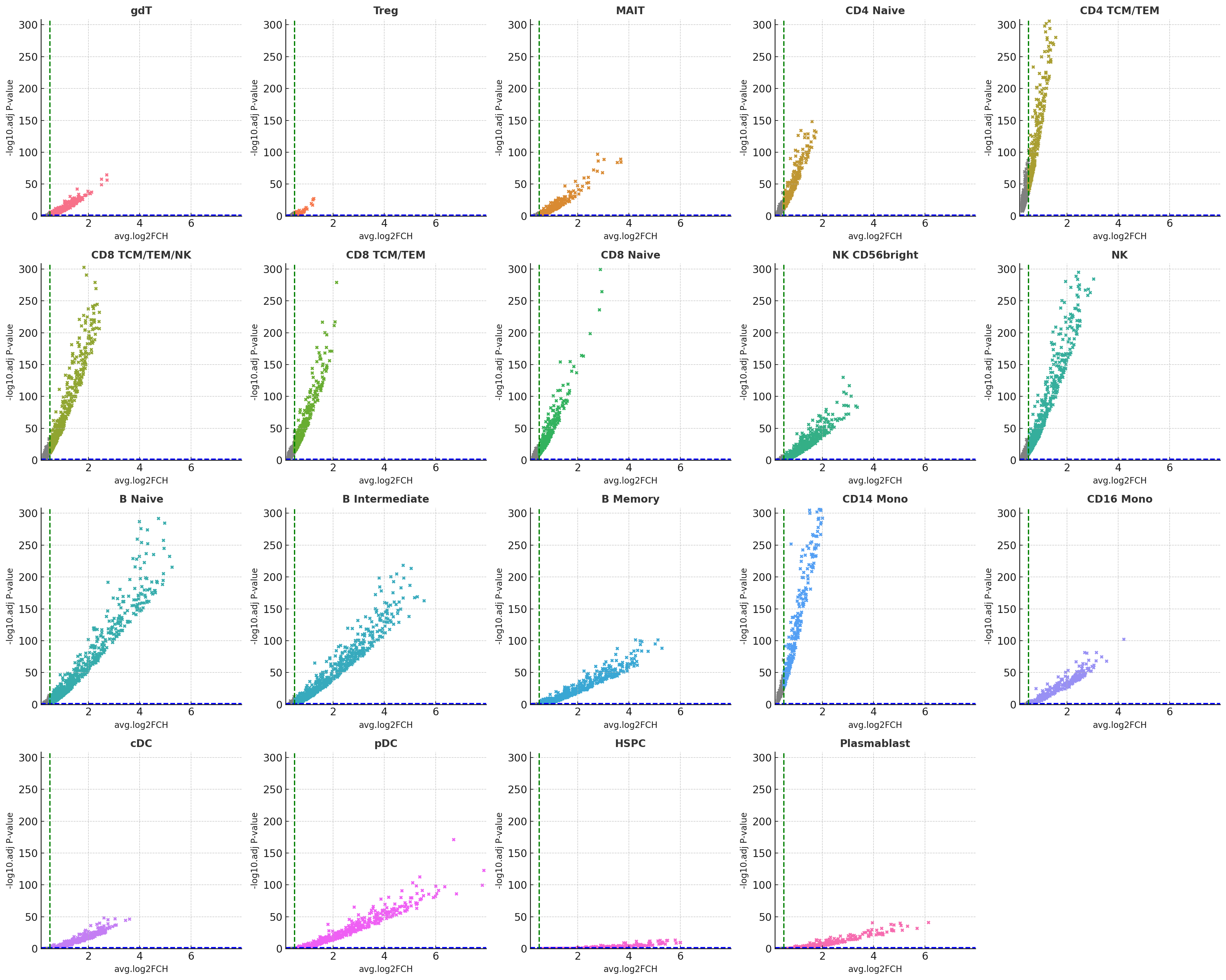
**Figure S8. Correlation Between Significance and ATAC-seq Log2 Fold Change Across Cell Subtypes**

This figure presents scatter plots illustrating the relationship between the significance (log10 adjusted p-value) and the average log2 fold change (avg.log2FC) of ATAC-seq peaks across different immune cell subtypes. Each panel corresponds to a specific cell subtype, including gd T, Treg, MAIT, *CD4*^+^ naïve T cells, *CD4*^+^ TCM/TEM, *CD8*^+^ TCM/TEM/NK, *CD8*^+^ TCM/TEM, *CD8*^+^ Naive, NK *CD56^bright^*, NK, B naive, B Intermediate, B Memory, CD14^+^ Monocytes, *CD16*^+^ Monocytes, cDC,s pDCs, HSPC, and Plasmablasts. The plots highlight significant changes in chromatin accessibility that correlate with gene expression, providing a visual representation of the epigenetic alterations across these immune cell subtypes.

**Figure S9.**

| 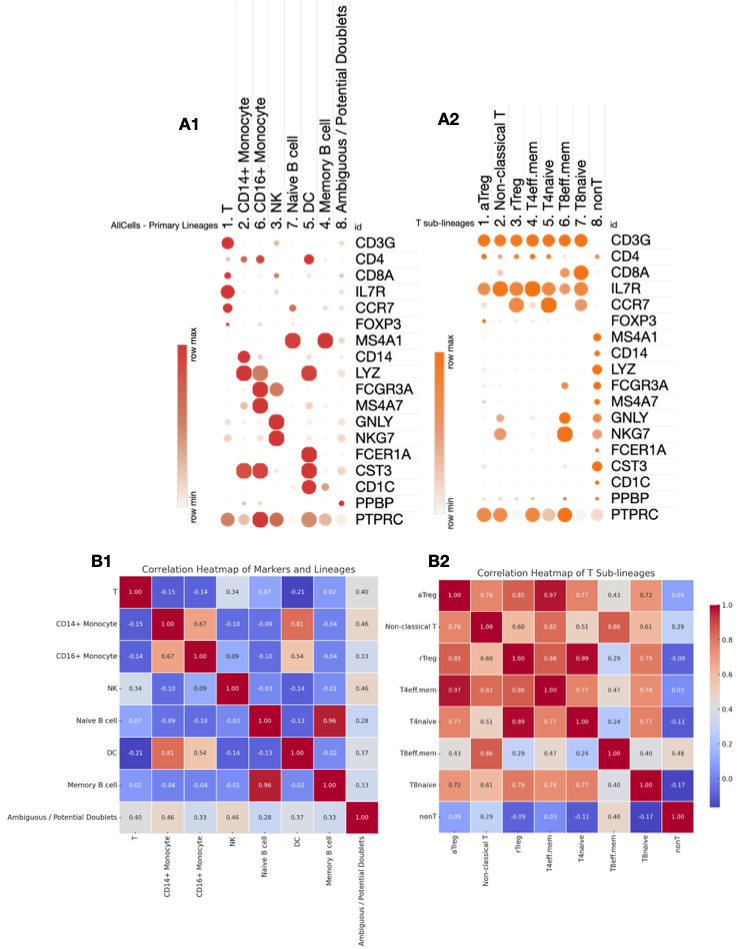 |
| --- |

****Figure S9.** Cell Marker Expression in Single-Cell RNA Sequencing of Peripheral Blood Mononuclear Cells (PBMCs) from Healthy Donors in the Immune Cell Atlas**

This figure illustrates the expression profiles of cell markers in PBMCs derived from healthy donors, as analyzed in the Immune Cell Atlas, available at the Single Cell Portal ****(https://singlecell.broadinstitute.org/single_cell)****

This figure presents two dot plots derived from single-cell RNA sequencing (scRNA-seq) data of PBMCs obtained from two healthy donors. The samples were processed using the 10X Genomics platform at two sites: the Broad Institute in Boston and Mount Sinai in New York City. The datasets were integrated employing Canonical Correlation Analysis (CCA) as described by Butler et al. Subsequent cell-type assignments were conducted through a hierarchical approach: **Primary Immune Lineage Classification**: Cells were initially categorised into primary immune lineages by fitting their expression profiles using multiple regression and naive Bayes optimisation, referencing gene lists from the Immune Cell Atlas (ICA). This process included manual curation to enhance accuracy. **Sub-lineage Assignment**: Within each primary lineage, cells were further classified into sub-lineages (e.g., Naïve CD4+ T cells) based on correlation with reference cell sets.

****Panel A1-2****: Dot plot illustrating the expression levels of selected marker genes across various primary immune lineages. The size of each dot represents the proportion of cells expressing the gene, while the colour intensity indicates the average expression level.

****Panel B1-2****: Dot plot depicting the expression patterns of marker genes across sub-lineages within a specific primary immune lineage. This visualisation aids in distinguishing functional subsets and understanding the heterogeneity within immune cell populations.

These visualisations provide insights into the cellular composition and gene expression dynamics of PBMCs in healthy individuals, serving as a reference for comparative studies in disease contexts.

**Figure S10.**

| 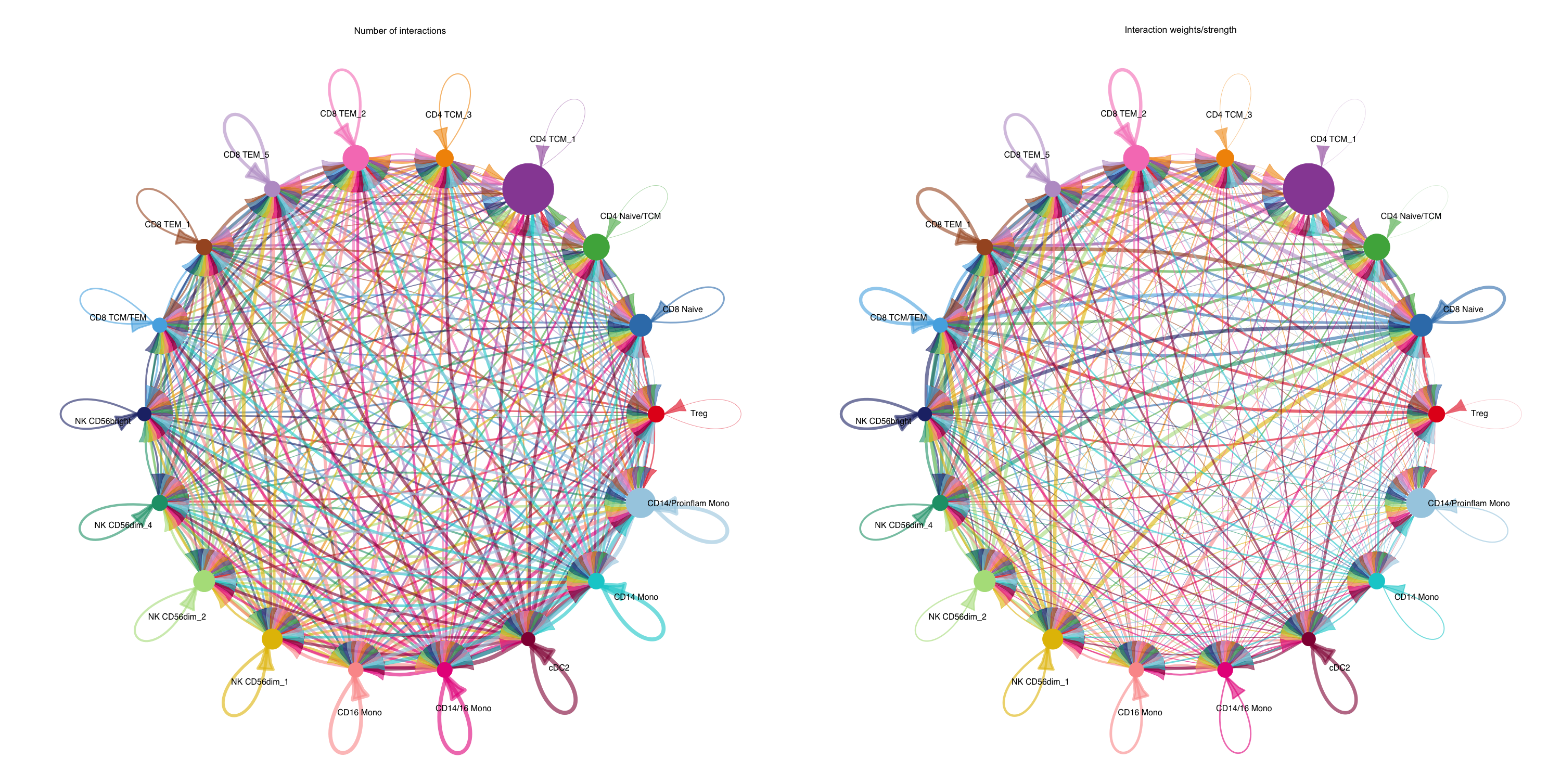 |
| --- |

**Figure S10. Aggregated cell–cell communication network in alopecia areata.** Global overview of the inferred intercellular communication network among immune cell subtypes in peripheral blood mononuclear cells (PBMCs) from alopecia areata patients. **Left panel:** Total number of inferred interactions between cell types, with edge thickness proportional to interaction count. **Right panel:** Interaction strength based on aggregated communication probability (weight), with edge thickness representing communication intensity.
Node size reflects the overall sending (outgoing) signal activity of each cell type. Self-loops indicate autocrine signaling potential.

**Figure S11.**

| 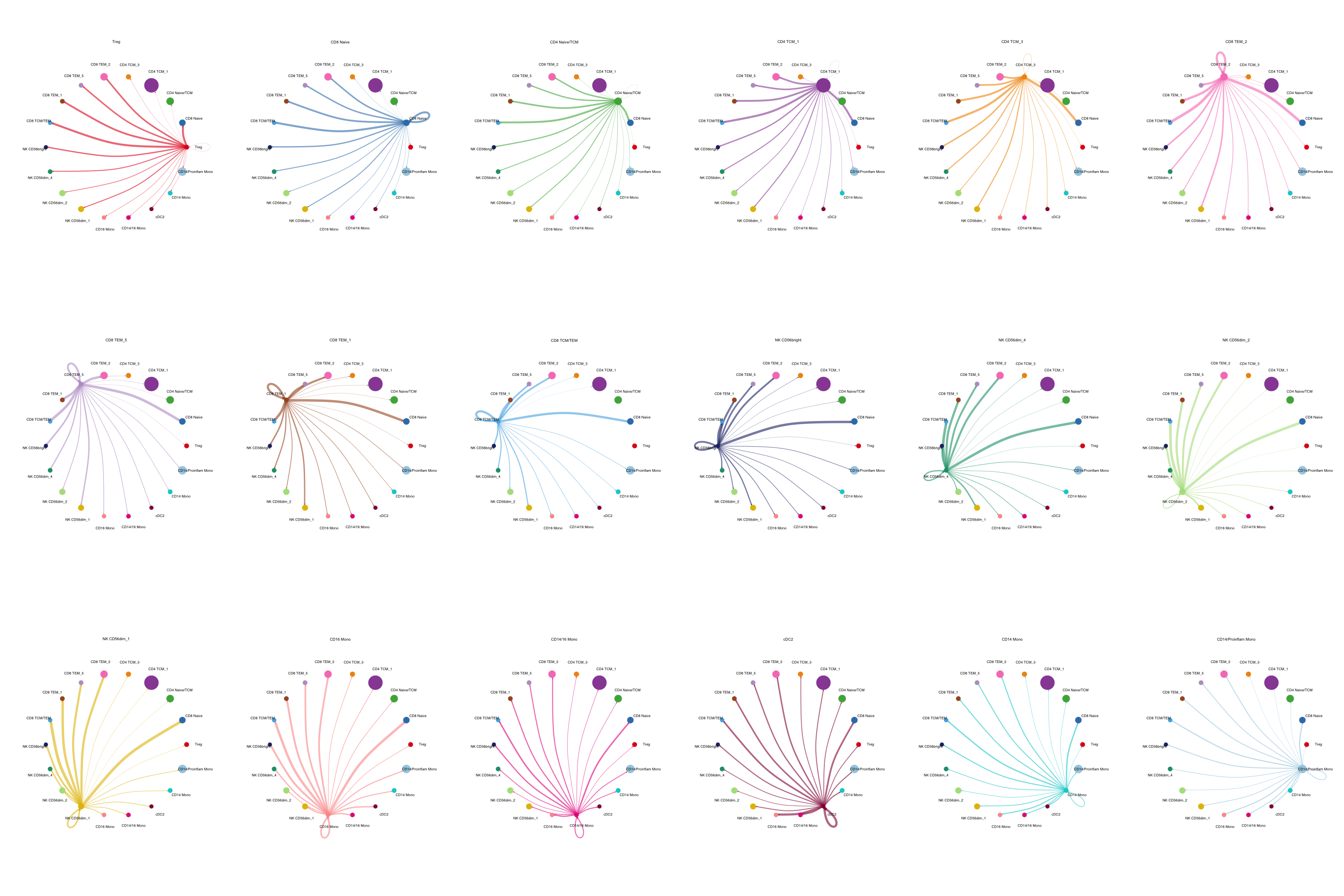 |
| --- |

**Figure S11. **Outgoing signalling patterns from individual cell subtypes.**** Outgoing communication networks originating from each individual immune cell subtype. Each circular panel represents the pattern of signalling sent from one specific cell population to all other detected subtypes. Arrow width represents the number and strength of significant ligand–receptor interactions. This analysis reveals the dominant sender cell types and their specific interaction targets in the immune microenvironment of alopecia areata.

**Figure S12.**

| 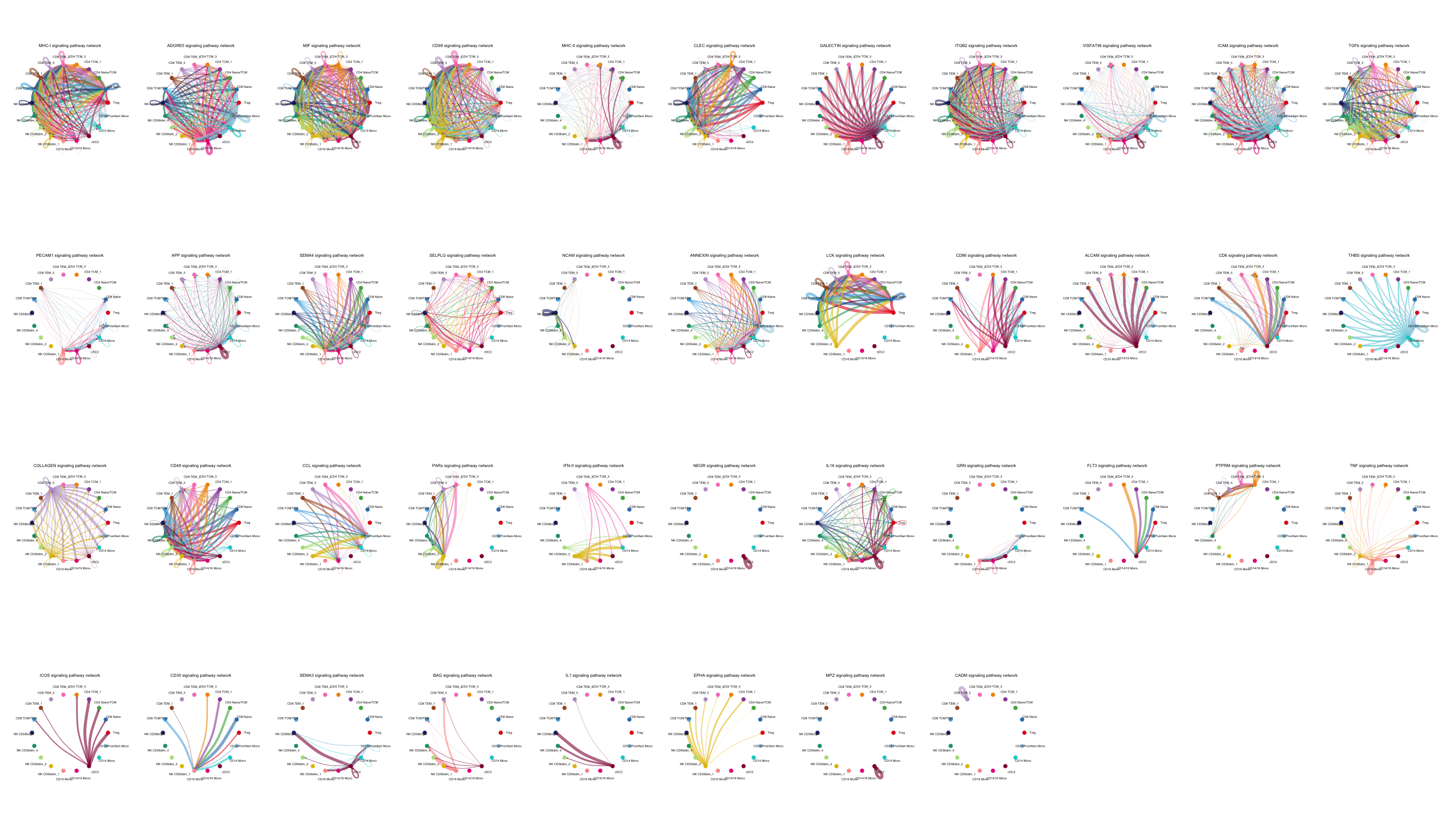 |
| --- |

### ****Figure S12. Contribution of cell subtypes to specific signalling pathways.** Cell-type–specific contribution to individual signalling pathways. Each circular diagram represents a distinct signalling pathway, with edges connecting sender and receiver cell types. The direction and colour of arrows reflect the pathway source and target populations, while edge width indicates the magnitude of signalling contribution. This figure highlights the functional specialisation of immune cell populations in mediating key immune signalling axes in alopecia areata.**
